# Six-year follow-up of participants in two clinical trials of rituximab or cyclophosphamide in Myalgic Encephalomyelitis/Chronic Fatigue Syndrome

**DOI:** 10.1371/journal.pone.0307484

**Published:** 2024-07-23

**Authors:** Ingrid G. Rekeland, Kari Sørland, Lisbeth Lykke Neteland, Alexander Fosså, Kine Alme, Kristin Risa, Olav Dahl, Karl J. Tronstad, Olav Mella, Øystein Fluge

**Affiliations:** 1 Department of Oncology and Medical Physics, Haukeland University Hospital, Bergen, Norway; 2 Department of Clinical Sciences, University of Bergen, Bergen, Norway; 3 Department of Oncology, Norwegian Radium Hospital, Oslo University Hospital, Oslo, Norway; 4 KG Jebsen Centre for B-Cell Malignancies, University of Oslo, Oslo, Norway; 5 Department of Biomedicine, University of Bergen, Bergen, Norway; Medizinische Universitat Innsbruck, AUSTRIA

## Abstract

**Objectives:**

In this six-year follow-up study, we used patient-reported outcome measures (PROMs) to compare values at baseline, at 18 months, and at six-year follow up from the CycloME and the RituxME trials.

**Methods:**

Based on the hypothesis that ME/CFS in a subgroup of patients is a variant of an autoimmune disease, we performed two clinical trials between 2014 and 2017. The RituxME trial was a randomized, double-blind and placebo-controlled phase III trial of 151 patients, assessing the B-cell depleting antibody rituximab. The CycloME trial was an open-label phase II trial of 40 patients using intravenous cyclophosphamide. Here we report six-year follow-up from both trials, using the Short Form 36 Physical Function (SF-36 PF) and DePaul short form (DSQ-SF) questionnaires.

**Result:**

Of the patients available after six years, 75.7% of RituxME and 94.4% of CycloME patients participated. In the RituxME rituximab group, the mean SF-36 PF scores were 32.9 at baseline, 42.4 at 18 months and 45.5 at six years. In the placebo group, the mean SF-36 PF scores were 32.3 at baseline, 45.5 at 18 months and 43.1 at six years. In the CycloME trial, mean SF-36 PF increased from 35.4 at baseline to 54.4 at 18 months, and 56.7 at six years. At six-year follow-up, 44.1% of cyclophosphamide-, 27.6% of rituximab- and 20.4% of placebo-treated patients had an SF-36 PF ≥ 70, and further, 17.6%, 8.6% and 7.4% of the corresponding patient groups had an SF-36 PF ≥ 90, which is within normal range. In terms of worsening at six years, 5.9% of cyclophosphamide-treated, 10.3% of rituximab-, and 14.8% of placebo-treated patients had a drop in SF-36 PF of 20 points or more from baseline. There were no serious unexpected adverse reactions.

**Conclusions:**

After six years, 44.1% of the cyclophosphamide group scored an SF-36 PF of at least 70, and 17.6% of at least 90, suggesting that cyclophosphamide in a subgroup may modulate the disease course in a beneficial way. However, cyclophosphamide carries toxicity concerns and should not be used for ME/CFS patients outside clinical trials. Rather, these data should encourage efforts to better understand the disease mechanisms and to search for targeted and less toxic immune modulatory treatment for this patient group.

## Introduction

Myalgic Encephalomyelitis/Chronic Fatigue Syndrome (ME/CFS) is a disease characterized by high symptom burden [1, 2], with profound impact on quality of life for patients and their caretakers [1–3], as well as high socio-economic costs [4]. Using the Canadian consensus criteria [5], ME/CFS is estimated to affect 0.1–0.8% of the population [6–9]. ME/CFS is preceded by an infection in 70%, with Epstein-Barr virus recognized as a common trigger [10]. The Covid-19 pandemic has further resulted in a large group of patients with similar symptoms (“Long Covid”), where studies have shown that a subgroup fulfills criteria for ME/CFS [11, 12]. The core symptoms of ME/CFS are severe fatigue and post-exertional malaise (PEM), cognitive disturbances, orthostatic intolerance and dizziness, sleep disturbances with inadequate restitution and pain. In addition, multiple symptoms may vary among patients. ME/CFS is a disease with unknown etiology, no validated biomarker, and no approved effective treatment. The British National Institute for Health and Care Excellence (NICE) recently published their revised guidelines for health workers [3], and the European Network on ME/CFS (EUROMENE) has also issued expert consensus recommendations on the diagnosis and care of patients [13].

Our interest in ME/CFS started in 2007, after incidental observations of patients with long-standing ME/CFS who got cancer, and who independently reported that the cancer treatment had beneficial effects on their ME/CFS disease. The treatments these patients had received included the cytotoxic drug cyclophosphamide or rituximab, a monoclonal B-cell depleting anti-CD20 antibody. These observations led to the hypothesis that ME/CFS in a subgroup of patients could be a variant of an autoimmune disease, often with a post-infectious onset, and with a role for B cells/plasma cells and antibodies. To pursue this hypothesis, we conducted clinical trials in patients with ME/CFS using the immunomodulatory drugs rituximab and cyclophosphamide [14–18]. The first studies with rituximab showed promising results. However, in a subsequent national, five-centre, double-blind and placebo-controlled phase III study of rituximab versus placebo (RituxME), there were no significant differences between the groups in any of the main outcomes measured [15]. Factors that may have influenced the negative result of the trial include heterogeneity among patients, placebo mechanisms, natural symptom variation, suboptimal outcome measures and lower maintenance doses of rituximab than in the previous phase II studies. Nevertheless, our pooled experience supports that some ME/CFS patients seem to respond to rituximab and B-cell depletion. If the autoimmunity hypothesis is correct, patients whose autoantibody production occurs in CD20 positive plasmablasts are likely responders to rituximab, as the drug specifically targets CD20 positive cells. In other patients, if the putative autoantibodies are produced in CD20 negative, long-lived plasma cells, rituximab is less likely to have clinical effect. At present we have no accurate method of identifying patients likely to respond to rituximab, and based on current evidence, rituximab cannot be recommended to ME/CFS patients selected by the Canadian consensus criteria alone [5].

In an open-label phase II trial with intravenous cyclophosphamide, half the patients reported clinical responses, which for the majority of responders lasted through three years follow-up [14]. Cyclophosphamide is a cytotoxic drug with broad effects on several subsets of lymphocytes, and beneficial mechanisms in ME/CFS could involve the anti-proliferative effects inhibiting B-cell activation to plasmablasts as well as altered maturation of B cells to plasma cells. Although acute toxicity in the CycloME trial was moderate, with nausea and general malaise after infusions being the most common side effects, cyclophosphamide carries a risk of side effects including infertility. A broader use in ME/CFS patients could thus be problematic due to toxicity concerns.

There is sparse literature describing the disease course of ME/CFS and prognosis over time, both for patients enrolled in clinical trials, and for patients in observational studies with no intervention. A study including 150 patients investigating onset and disease course in ME/CFS found that 59% described a fluctuating course over time, while 4% reported steadily improving symptoms [19]. However, the prevalence of several fatigue-related symptoms was less pronounced at the time of survey compared to patient recalling of symptoms from the initial six months after disease onset. A recent retrospective study described 168 patients with ME/CFS in an outpatient clinic in France, included between 2011 and 2019 with follow-up until December 2020. The authors reported full recovery in 8.3% and improvement in 4.8%, in total 13.1%, and concluded that the prognosis is generally poor [20].

A systematic review from 2005 included 14 studies of participants with CFS and found that a median 5% had a full recovery, while a median 39.5% experienced an improvement over a time span of 1 to 5 years [21].

In 2020, the Norwegian ME association performed an internet-based questionnaire survey of ME/CFS patients. The 5822 participants classified the course of their disease up to the time of participation: 12% reported improvement, 23% a stable course, 29% large variations, and 35% worsening. Only 2% reported full recovery. Irrespective of overall pattern of disease, the majority described a disease course characterized by variation over time [22]. However, it is possible that such online surveys underestimate the degree of recovery over time, as patients who recovered may be less interested in following the ME/CFS-related internet pages and social media groups used to promote the survey.

Here we report results from follow-up studies of patients included in the RituxME and CycloME intervention trials, at least six years after inclusion. Specifically, we wanted to explore the long-term course of disease symptoms and possible late side effects of treatment. Since the trials were similar with regards to time period of inclusion, inclusion criteria and outcome measures, we wanted to compare long-term outcome in the two trials.

## Methods

### Participants

**Fig 1:**
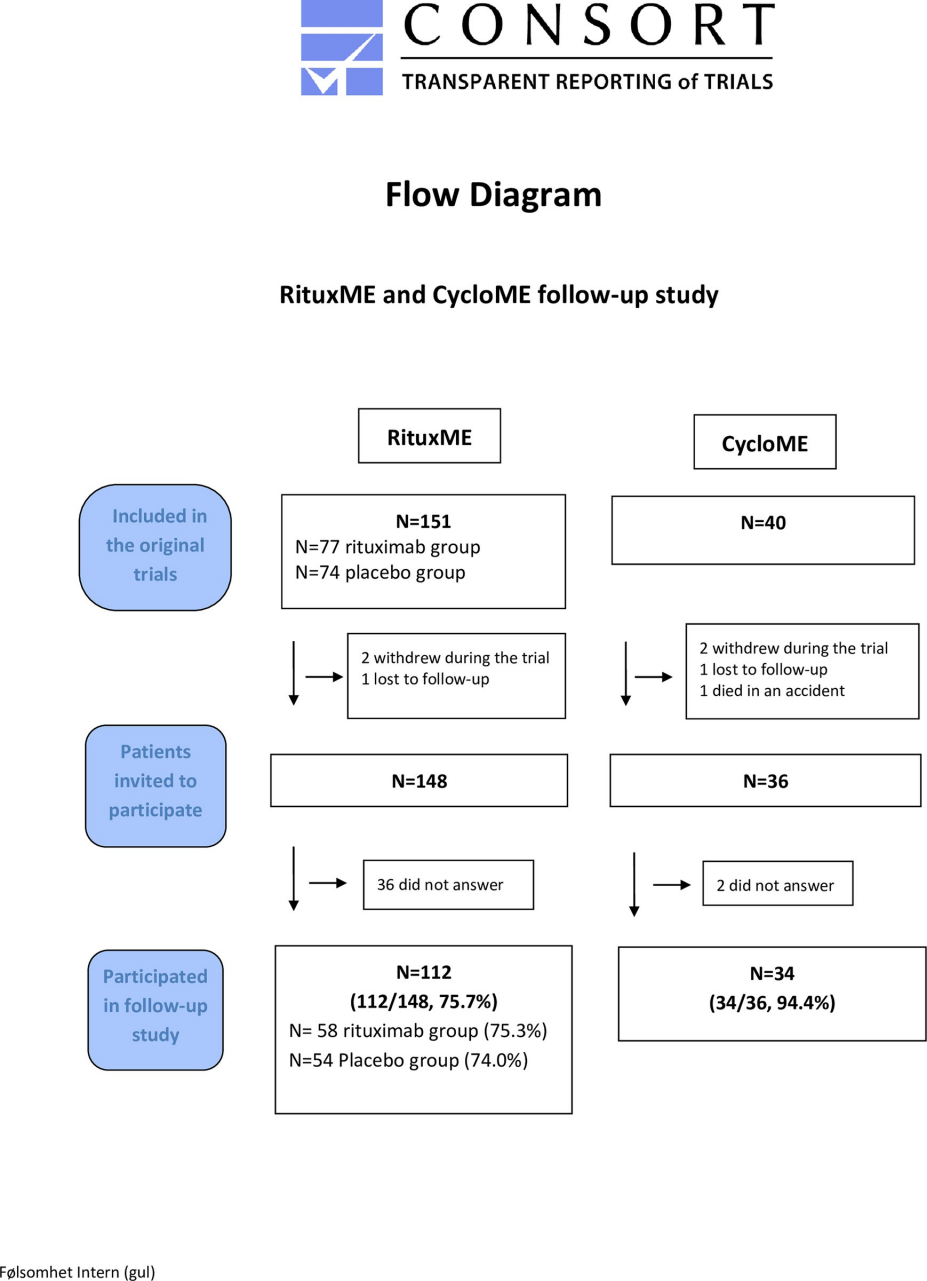
CONSORT flow diagram. Flow diagram of invited and included patients in the six-year follow-up study of the RituxME and CycloME trials.

The follow-up study for RituxME was performed from 01.05.2021 to 01.09.2021, six years after inclusion. The follow-up study for CycloME was performed from 25.10.2022 to 31.01.2023, six to seven years after inclusion. The participants received questionnaires by mail and returned completed questionnaires to the study coordinator at Haukeland University Hospital. The participants were registered by their study-specific number. Details on inclusion, outcome measures and follow-up in the study period have been reported previously for both the RituxME [15] and CycloME [14] trials. All patients fulfilled the Canadian Consensus criteria [5] after clinical assessment, before inclusion in the trials in 2014/15. The RituxME trial included 151 patients: 77 in the rituximab and 74 in the placebo group. Two withdrew during the trial, and one was lost to follow-up. Out of the 148 remaining patients, all were invited and 112 (75.7%) participated in the six-year follow-up study. In the CycloME trial, 40 patients were included; two withdrew during the study treatment period, one was lost to follow-up, and one died in an accident. Out of the 36 remaining patients, 34 (94.4%) participated in the six-year follow-up study. Fig 1 is a flowchart of participants in the follow-up studies for both the RituxME and CycloME trials. The supplementary files include the protocols for the two trials, with amendments describing the six-year follow-up studies (S4, S5 Files).

The RituxME trial, which was a national, five-center, randomized, and double-blind phase III trial, included patients from 2014 to 2015. The patients received either rituximab or placebo, initially two intravenous infusions (500 mg/m^2^, max 1000 mg) two weeks apart, then maintenance treatment with reduced fixed doses of 500 mg at three, six, nine and 12 months. The primary endpoint for response was evaluated after 18 months, and patients were monitored for a total of 24 months.

In the CycloME trial, which included patients in 2015 and 2016, patients received six infusions of cyclophosphamide intravenously every four weeks, 600 mg/m^2^ at first dose, then 700 mg/m^2^ for the further five doses. The trial was single-site. All patients were included at HUS, and seven patients received parts of their treatment and follow-up at the Department of Oncology, Oslo University Hospital (OUH). Follow-up initially lasted until 18 months, with an extra visit at three to four years following a protocol amendment.

The inclusion criteria for the RituxME and CycloME studies were similar, with some exceptions. In the RituxME trial, patients with mild severity and a disease duration of at least five years were eligible for inclusion, while patients with mild ME/CFS were not included in the CycloME trial. The RituxME trial did not include patients with ME/CFS disease duration of more than 15 years, while the CycloME trial had no upper limit for disease duration. Selected baseline characteristics of participants in the RituxME and CycloME trials and in the present six-year follow-up study are shown in Table 1.

**Table 1:** Baseline data and data from six-year follow-up.

	CycloME	RituxME	
	Cyclophosphamide iv	Rituximab iv	Placebo iv
Included in trial 2014/15, N	40	77	74
Completed original trial^1^, N	38	75	73
Invited for 6-y follow-up study	36	75	73
Participants at 6-y follow-up^2^	34	58	54
Sex, female, N (%)	26/34 (76)	48/58 (83)	48/54 (89)
Age at inclusion, mean (SD)	41.4 (10.4)	38.2 (12.2)	36.2 (11.3)
Steps/24h baseline, mean (SD)	3331 (2068)	3042 (1799)	2996 (2028)
ME/CFS severity, baseline, N (%) ^3^			
Mild	0	8/57 (14)	5/54 (9)
Mild/moderate	14/34 (41)	13/57 (22)	17/54 (31)
Moderate	11/34 (32)	16/57 (28)	14/54 (26)
Moderate/severe	6/34 (18)	9/57 (16)	10/54 (19)
Severe	3/34 (9)	11/57 (19)	8/54 (15)
ME/CFS duration, baseline, N (%)			
2–5 years	6/34 (18)	9/58 (16)	12/54 (22)
5–10 years	12/34 (35)	31/58 (53)	32/54 (59)
10–15 years	8/34 (24)	18/58 (31)	10/54 (19)
>15 years	8/34 (24)	0	0
Clinical response 18m, N (%)^4^	20/34 (59)	16/58 (28)	19/54 (35)
No clinical response 18m, N (%)^4^	14/34 (41)	42/58 (72)	35/54 (65)
**PROMS, N**	34	58	54
SF-36 Physical Function ^5^			
Baseline, mean (SD)	35.4 (17.4)	32.9 (22.3)	32.3 (16.9)
18 months	54.4 (27.4)	42.4 (25.0)	45.5 (26.1)
6 years	56.7 (27.6)	45.5 (29.5)	43.1 (28.6)
SF-36 PF, mean (SD) ^6^			
Difference 6y –baseline	21.2 (27.7)	12.6 (27.7)	10.7 (28.4)
Difference 6y - 18m	2.3 (21.9)	3.1 (20.9)	-2.4 (17.5)
SF-36 PF at 6y, N (%) ^7^			
SF-36 SF ≥ 40	22/34 (64.7%)	30/58 (51.7%)	28/54 (51.9%)
SF-36 PF ≥ 50	18/34 (52.9%)	29/58 (50.0%)	25/54 (46.3%)
SF-36 PF ≥ 60	18/34 (52.9%)	22/58 (37.9%)	20/54 (37.0%)
SF-36 PF ≥ 70	15/34 (44.1%)	16 /58 (27.6%)	11/54 (20.4%)
SF-36 PF ≥ 80	7/34 (20.6%)	10/58 (17.2%)	7/54 (13.0%)
SF-36 PF ≥ 90	6/34 (17.6%)	5/58 (8.6%)	4/54 (7.4%)
DSQ_SF ^8^			
DSQ total score baseline, mean (SD)^8^	72.9 (12.7)	NA	NA
DSQ Canadian criteria baseline, N (%)^9^	33/34 (97.1)	NA	NA
DSQ total score at 6y, mean (SD)	50.8 (22.5)	54.0 (22.8)	60.1 (20.6)
DSQ Canadian criteria at 6y, N (%)^9^	18/34 (52.9)	34/58 (58.6)	37/54 (68.5)
Function level ^10^			
Baseline, mean (SD)	17.7 (7.2)	18.8 (10.8)	17.9 (8.0)
18 months	36.0 (26.0)	24.6 (17.8)	25.3 (17.9)
6 years	38.4 (28.7)	30.9 (24.8)	28.6 (21.3)

^1^: number of patients completing 18 months follow-up in the CycloME trial, and 24 months in the RituxME trial

^2^: Participants at six years’ follow-up, out of trial patients invited

^3^: ME/CFS severity baseline, one missing data in the rituximab group, n = 57.

^4^: Clinical response at 18 months according to the primary endpoint in the original trials

^5^: Mean SF-36 PF (scale 0–100, higher number denotes better function) at baseline, 18 months and six years

^6^: SF-36 PF mean difference between six years and baseline, and between six years and 18 months

^7^: Number of patients with SF-36 PF at least 40, 50, 60, 70, 80 and 90 points at six years

^8^: DePaul Symptom Questionnaire, Short Form. NA, not applicable, not performed at baseline in the RituxME trial

^9^: Fulfilled Canadian criteria, according to diagnostic algorithm in the DSQ-SF questionnaire

^10^: Mean Physical Function, scale 0–100, according to a table with examples. Missing data from one patient in the rituximab group at baseline, and two patients from the placebo group; one at baseline and one at 18 months.

### Objectives

In this six-year follow-up study, we used patient-reported outcome measures (PROMs). We compared values at baseline, at 18 months, and at six-year follow up for the CycloME trial and RituxME trial (rituximab and placebo groups).

### Patient-reported outcome measures

For health-related quality of life, we used the validated Norwegian translation of the Short Form 36 Health Survey ver. 1.2 (SF-36) [23]. A Norwegian version of the DePaul Symptom Questionnaire—Short Form (DSQ-SF) [24] based on the validated translation of the relevant items in the complete DePaul Symptom Questionnaire [25] captured ME/CFS symptoms (English version, S1 File).

The SF-36 contains 36 items on health-related quality of life. We used the SF-36 domains Physical Function, Bodily Pain, General Health, Vitality, Social Function and Mental Health (raw scores, scale 0–100) and focused on Physical Function (PF). The normal range for SF-36 PF in the population varies with age and sex, with higher scores in younger age groups and lower scores (indicating lower physical function) in women [26].

DSQ-SF examines the frequency and severity of 14 typical ME/CFS symptoms during the previous six months. Higher scores indicate higher symptom burden (score 0–112). A predefined algorithm estimates whether the patients fulfill the Canadian diagnostic criteria for ME/CFS. We applied this algorithm in the six-year follow up. For the CycloME patients, compliance with Canadian criteria was compared with baseline values. For the RituxME patients, this comparison was not possible, as the DSQ-SF was not administered at baseline.

### Function level and study specific questionnaire

The patients received a questionnaire recording self-reported Function level (%) from 0–100% according to a table with examples and items related to general health, to their ME/CFS disease and possible interventions undertaken. These items included assessment of health status at 6 years compared to end of study (healthier, no change, worse); any new diagnoses after end of study; any specific treatment aimed at ME/CFS after end of study; any changes in diet after end of study; whether any treatment or other measures have affected the ME/CFS disease after end of study (S1 File).

### Statistics

Descriptive methods were used to characterize the patient sample by treatment groups (cyclophosphamide, rituximab, placebo). Differences between the treatment groups in repeated measures of outcomes were assessed by General Linear Model (GLM) for repeated measures, using the time-by-treatment group interaction. For comparison of outcomes (SF-36 PF and Function level) over time (baseline, 18 months, six years), by treatment groups (cyclophosphamide, rituximab, placebo), adjustments were made for age, sex, study centre and baseline ME/CFS severity (included in the model as covariates). Greenhouse-Geisser corrections were used for all GLM analyses due to violations of the sphericity assumption.

Logistic regression was performed (method backwards stepwise) with SF-36 PF at six years (< 70 versus ≥ 70) as the dependent variable, and with age, sex, study centre, baseline SF-36 PF and clinical trial as predictor variables. The cutoff value of SF-36 PF 70 was used because it reflects a significant improvement in function from the mean baseline level. The cutoff value was not predefined in the protocol. All tests were two-sided, and the significance level was set to 0.05. All analyses were performed using IBM SPSS Statistics ver.28 (IBM Corp., Armonk, NY), and Graphpad Prism ver.9 (GraphPad Software, La Jolla, CA).

The syntax for GLM repeated measures and for logistic regression analyses are shown in supplementary data; S7 File SPSS Analysis code.

### Ethics

The studies for six-year follow-up were approved by the Regional Committees for Medical and Health Research Ethics in Norway, as amendments to the two clinical trials; RituxME: 2014/365, CycloME: 2014/1672 (S6 File). The original trials are registered in ClinicalTrials.gov; for CycloME: NCT02444091, and for RituxME: NCT02229942.

## Results

### Participation in the six-year follow-up study

Among the RituxME study patients, 112 out of 148 eligible patients (75.7%) participated; 77.3% of the rituximab- and 74.0% of the placebo group (Table 1, and Fig 1). Of the 148 eligible patients, 16 out of 26 men (61.5%), and 96 out of 122 women (78.7%) participated in the six-year follow up. For the RituxME cohort, patients with severe ME/CFS at baseline had a 90% rate of participation in the six-year follow-up, compared to 73% in those with mild and mild/moderate disease. Out of patients with disease duration 10–15 years at baseline, 88% participated, as compared to 72% in patients with disease duration 2–10 years. For the CycloME trial, 34 out of 36 (94.4%) available patients at six years participated. One patient with severe and one with moderate disease did not participate. Participation among the patients fulfilling the response criteria in the CycloME study was 95% compared to 93% among the non-responders. In the RituxME rituximab group, 84% of the patients fulfilling the response criteria participated, compared to 75% among the patients with no clinical response. The placebo group had approximately the same participation rate for the patients with or without clinical response; 73% and 74%, respectively. Table 2 shows baseline characteristics and outcome measures at 18 months, comparing participants and non-participants in the six-year follow-up study of the CycloME and RituxME trials.

**Table 2:** Baseline characteristics and outcome measures at 18 months, comparing participants and non-participants in the six-year follow-up study of the CycloME and RituxME trials.

	CycloME		RituxME			
	Cyclophosphamide iv		Rituximab iv		Placebo iv	
Included in trial 2014/15, N	40		77		74	
Available for 6-y follow-up, N	36		75		73	
**6-y follow-up **	**Participants **	**Non-participants **	**Participants **	**Non-participants **	**Participants **	**Non-participants **
Participation, N (%)	34/36 (94)	-	58/75 (77)	-	54/73 (74)	-
Sex, female, N (%)	26/28 (93)	2/28 (7)	48/62 (77)	14/62 (23)	48/60 (80)	12/60 (20)
Sex, male, N (%)	8/8 (100)	0/8 (0)	10/13 (77)	3/13 (23)	6/13 (46)	7/13 (54)
Age at baseline, mean (SD)	41.4 (10.4)	50.9 (14.5)	38.2 (12.1)	36.8 (9.3)	36.2 (11.3)	34.0 (11.1)
*ME/CFS severity*, *baseline*, *N (%)*^*1*^^,^^*4*^						
Mild^2^	NA	NA	8/11 (73)	3/11 (27)	5/6 (83)	1/6 (17)
Mild/moderate	14/14 (100)	0	13/18 (72)	5/18 (28)	17/24 (71)	7/24 (29)
Moderate	11/12 (92)	1/12 (8)	16/21 (76)	5/21 (24)	14/22 (64)	8/22 (36)
Moderate/severe	7/7 (100)	0	9/12 (75)	3/12 (25)	10/12 (83)	2/12 (17)
Severe	2/3 (67)	1/3 (33)	11/12 (92)	1/12 (8)	8/9 (89)	1/9 (11)
*ME/CFS duration*, *baseline*, *N (%)*^*3*^						
2–5 years	6/6 (100)	0	9/11 (82)	2/11 (18)	12/17 (71)	5/17 (29)
5–10 years	12/13 (92)	1/13 (8)	31/44 (70)	13/44 (30)	32/44 (73)	12/44 (27)
10–15 years	8/8 (100)	0	18/20 (90)	2/20 (10)	10/12 (83)	2/12 (17)
>15 years^2^	8/9 (89)	1/9 (11)	NA	NA	NA	NA
Clinical response at 18m, N (%)	20/21 (95)	1/21 (5)	16/19 (84)	3/19 (16)	19/26 (73)	7/26 (27)
No clinical response at 18m, N (%)	14/15 (93)	1/15 (7)	42/56 (75)	14/56 (25)	35/47 (74)	12/47 (26)
SF-36 PF baseline mean (SD) (min-max)	35.4 (17.4) (0–65)	17.5 (24.7) (0–35)	32.9 (22.3) (0–75)	44.1 (20.0) (20–75)	32.3 (16.9) (0–80)	34.0 (24.7) (0–80)
SF-36 PF 18m, mean (SD) (min-max)	54.4 (27.4) (0–100)	37.5 (53.0) (0–75)	42.4 (25) (0–95)	56.5 (29.2) (0–90)	45.5 (26.1) (0–95)	45.5 (26.3) (0–85)
Physical function baseline (SD) (min-max)^5^	17.7 (7.2) (5–40)	17.5 (17.7) (5–30)	18.8 (10.8) (6–50)	25.2 (13.2) (10–60)	17.9 (8.0) (5–40)	20.3 (11.1) (7–50)
Physical Function 18m, (SD) (min-max)^6^	36.0 (26.0) (5–95)	21.5 (26.2) (3–40)	24.6 (17.8) (3–80)	29.9 (15.1) (9–70)	25.3 (17.9) (4–80)	27.3 (20.1) (3–70)

^1^ ME/CFS severity; CycloME trial did not include mild disease severity

^2^ NA: not applicable

^3^ ME/CFS duration; RituxME trial did not include patients >15 years disease duration

^4^ ME/CFS severity; rituximab group, n = 57, one participant with missing data

^5^ Physical function baseline; placebo group, one participant missing data

^6^ Physical function 18 months; placebo group; one participant missing data. CycloME; one non-participant missing data

### SF-36 Physical Function and DSQ-SF

During the first 18 months of the RituxME trial (rituximab and placebo groups pooled), patients reported an overall improvement with a mean increase in SF-36 PF of 11.5 (from 33.9 to 45.4), with no difference between the rituximab and placebo groups [15]. From 18 months in the RituxME trial until the six-year follow-up, there was no notable change in mean SF-36 PF; 42.4 to 45.5 in the rituximab group and 45.5 to 43.1 in the placebo group.

In the CycloME trial [14], there was a mean improvement in SF-36 PF of 19 points (from 35.4 to 54.4) during the initial 18 months of the study, including an increase of 34.5 points (from 35.0 to 69.5) among the 22 patients (55%) registered as responders to cyclophosphamide. For the 34 participants at six-year follow-up, there was a further slight increase in mean SF-36 PF of 2.3 (from 54.4 to 56.7) at six years. The mean SF-36 PF among the 20 patients categorized as responders in the CycloME trial was 70.3 at 18 months, and 67.4 at six years.

The mean values for all SF-36 domains–Physical Function, Bodily Pain, General Health, Vitality, Social Function and Mental Health–in the RituxME (rituximab and placebo groups) and CycloME trials, at baseline, 18 months and six years are shown in Supplementary data (S2 File). The raw data for PROMs from baseline and follow-up raw are shown in Supplementary data (S3 File).

**Fig 2:**
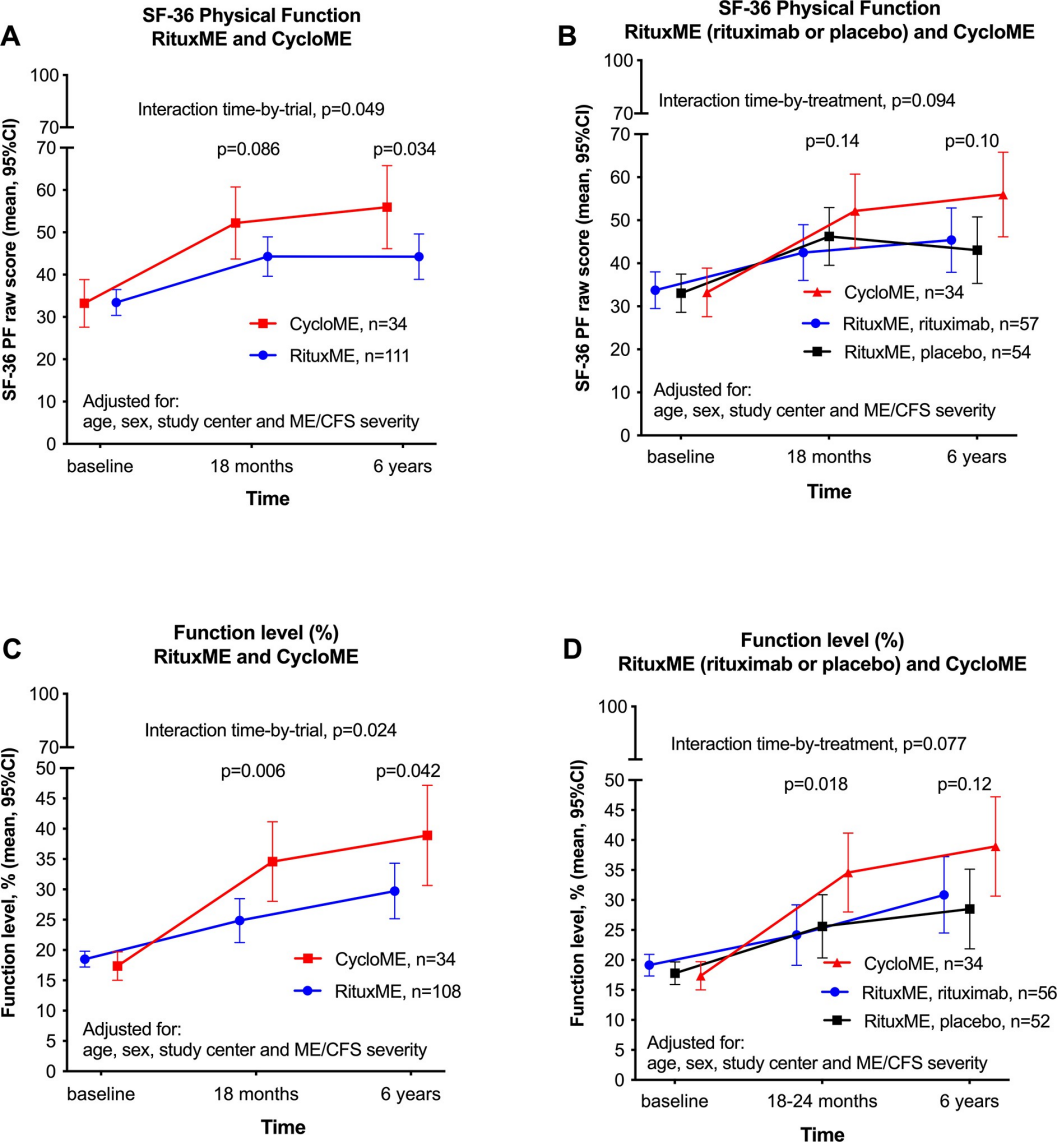
SF-36 Physical Function and Function level by trial and treatment. (A) and (B) show SF-36 Physical Function (mean, 95% CI) from baseline, 18 months and six years in (A) the RituxME and CycloME trials and (B) both trials, with the RituxME trial divided into treatment categories; placebo and rituximab groups. (C) and (D) show self-reported Function level (%) (mean, 95% CI) from baseline, 18 months and six years in (C) the RituxME and CycloME trials and (D) both trials, with the RituxME trial divided into treatment categories; placebo and rituximab groups. All four analyses–(A), (B), (C) and (D)–were performed by General Linear Model repeated measures, adjusted for age, sex, study centre and baseline ME/CFS severity, with p-values for the interaction time-by-treatment group. The p-values for each timepoint 18 months or 6 years, as compared to baseline, are from the simple contrasts in the time domain, for the interaction term. The colours indicate treatment groups, described in the Figure. In panel (A) and (B) RituxME n = 111, due to one missing severity grade registration in the rituximab group. In panel (C) and (D) n = 108, due to missing data of severity in one patient in the rituximab group, and missing data for function level in three different patients at one timepoint.

Given the similar inclusion criteria for the two intervention trials, we compared the courses of SF-36 PF using GLM repeated measures, adjusted for age, sex, study center and baseline ME/CFS severity. There was a significant difference in the course of SF-36 PF and of Function level in favor of the CycloME compared to RituxME participants over 6 years. In other words, the changes in SF-36 PF over time, as compared to baseline, were significantly different between the groups (Fig 2, panels A and C).

**Fig 3:**
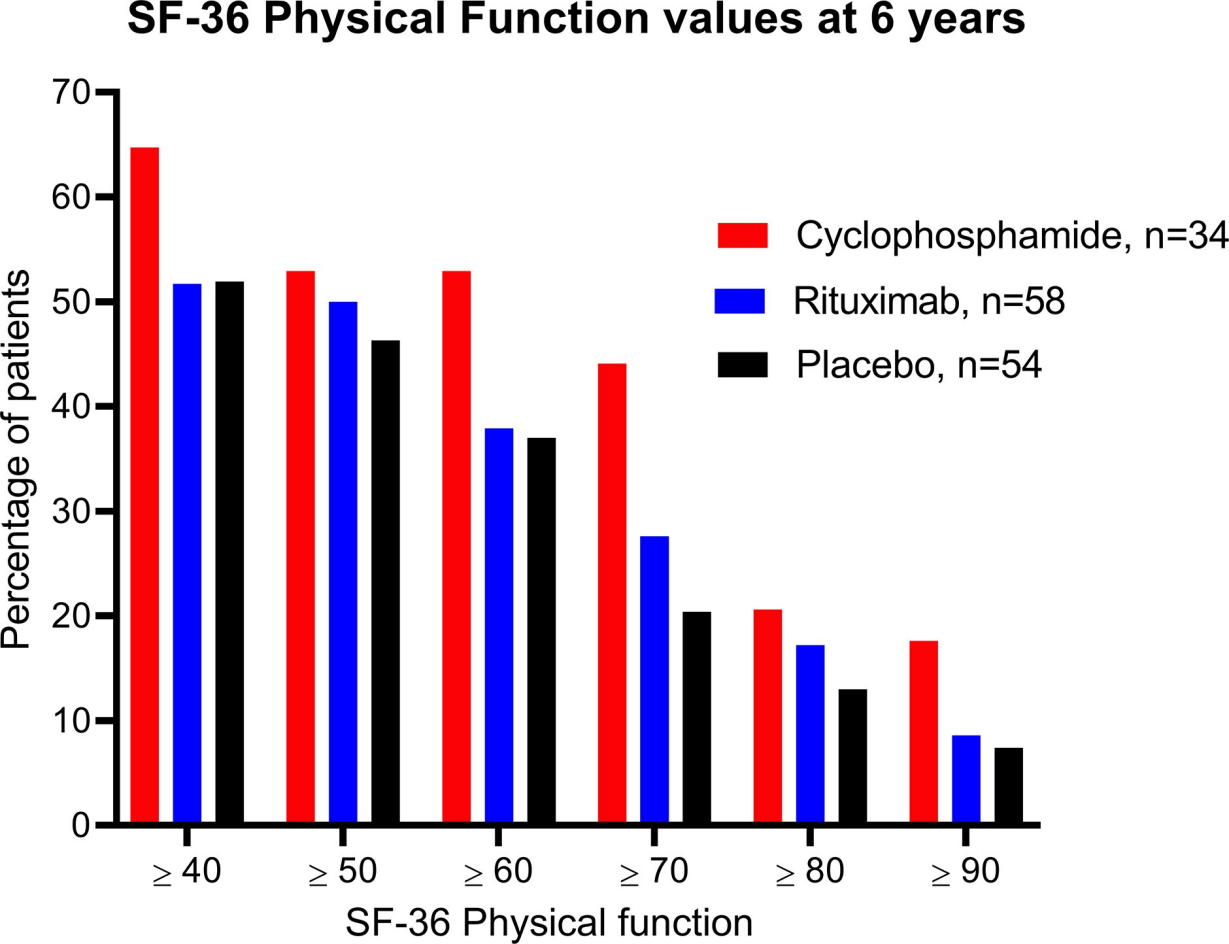
Percentages of patients with different SF-36 physical function scores at six years. Percentages of patients with different levels of SF-36 Physical Function values at six years. The three columns represent the CycloME trial participants and the rituximab and placebo groups from the RituxME trial. The colours indicate treatment groups, described in the Figure.

Fig 3 shows the percentages of patients in the three groups (cyclophosphamide, rituximab, placebo) who reached different cutoff levels of SF-36 PF at six years. In the cyclophosphamide group, 44.1% had SF-36 PF ≥ 70 at six years, compared to 27.6% in the rituximab group and 20.4% in the placebo group (Fig 3, panel A, Table 1). The difference between trials for SF-36 PF ≥ 70 was significant, i.e. 44.1% for CycloME versus 24.1% for RituxME (p = 0.024). At six years, 17.6% of the cyclophosphamide patients, 8.6% of the rituximab patients, and 7.4% of the placebo patients had SF-36 PF ≥ 90. The difference between trials for SF-36 PF ≥ 90 did not reach statistical significance, i.e. 17.6% for CycloME versus 8.0% for RituxME (p = 0.10).

**Fig 4:**
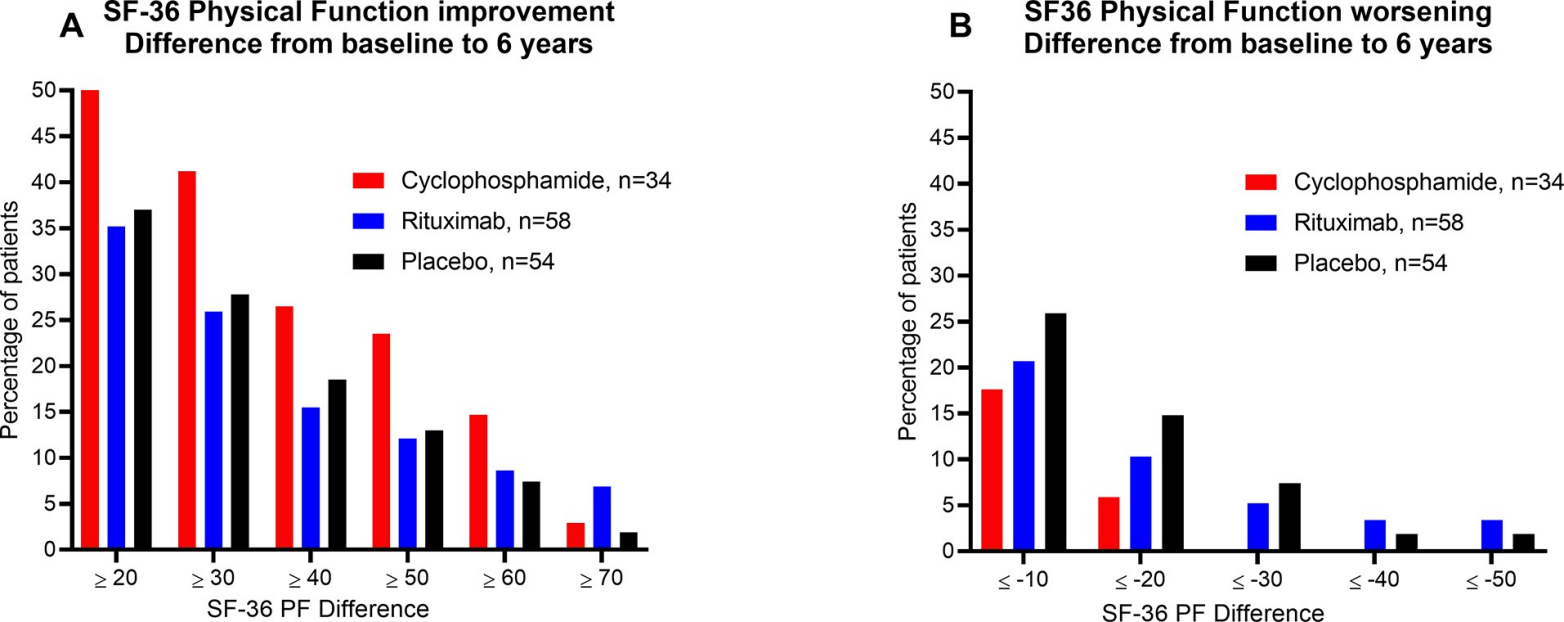
SF-36 Physical Function improvement and worsening, difference from baseline to 6 years. Percentages of patients who have experienced various degrees of change in SF-36 Physical Function from baseline to 6 years. The three columns represent the CycloME trial participants and the rituximab and placebo groups from the RituxME trial. (A) shows improvement and (B) shows worsening. The colours indicate treatment groups, described in the Figure.

In logistic regression analyses with SF-36 PF cutoff 70 as the dependent variable, with age, sex, baseline SF-36 PF, study centre and clinical trial included in the model, baseline SF-36 PF (p<0.001) and clinical trial (p = 0.022) were the significant independent variables; CycloME versus RituxME, odds ratio (OR) 2.77 (95% confidence interval (CI) 1.16 to 6.62). Assessing the differences between SF-36 PF at baseline and 6 years, 23.5% of the cyclophosphamide-, 12.1% of the rituximab-, and 13.0% of the placebo-treated patients had increased by 50 points or more (Fig 4, panel A). For worsening at 6 years follow-up, 5.9%, 10.3% and 14.8% of the respective groups experienced a drop in SF-36 PF of 20 points or more from baseline. None of the cyclophosphamide-, 5.2% of the rituximab-, and 7.4% of the placebo-treated patients reported a drop of 30 points or more (Fig 4, panel B).

**Fig 5:**
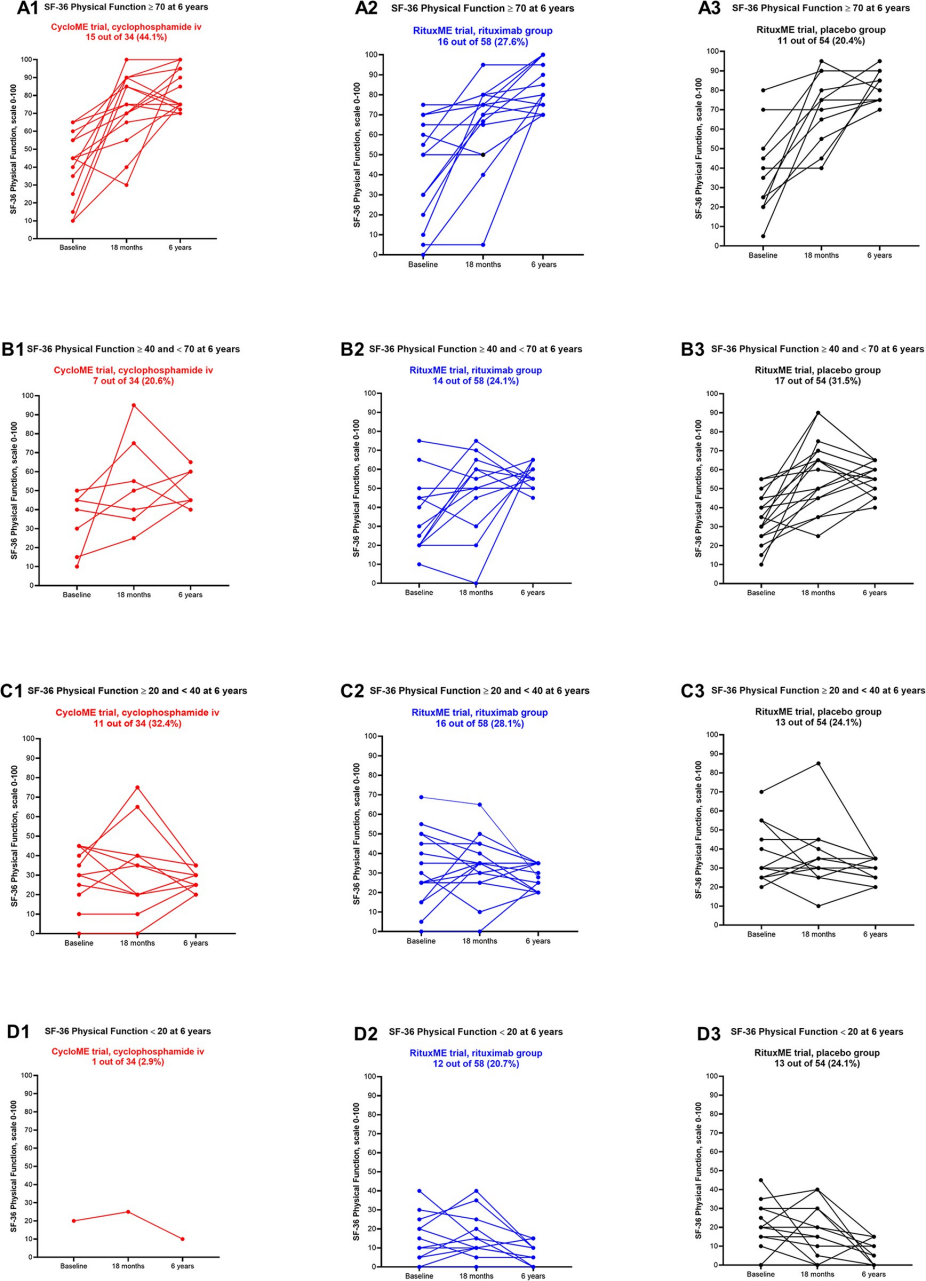
SF-36 Physical Function by treatment group and different values of SF-36 at six years. Fig 5 describes the individual courses of SF-36 Physical function, from baseline to 18 months and six years, by intervention group (1) CycloME, (2) RituxME rituximab group and (3) RituxME placebo group, and divided according to SF-36 PF score at six years; (A) more than 70, (B) between 40 and 70, (C) between 20 and 40, and (D) less than 20 points.

Fig 5 shows the individual courses of SF-36 PF, at baseline, 18 months and six years, by intervention group, and divided according to level of SF-36 PF at six years (more than 70, between 40 and 70, between 20 and 40, and less than 20). Many patients reported considerable variation over time, while others experienced a relatively stable course.

The DSQ-SF questionnaire was applied both at baseline and at six-year follow-up in the CycloME trial, but only at the six-year follow-up in the RituxME trial. In the cyclophosphamide group, the baseline mean DSQ-SF score was 72.9 (SD 12.7), while at six-year follow-up the mean DSQ-SF score was 50.8 (SD 22.5.0) (p<0.001). In the cyclophosphamide group, according to the DSQ-SF algorithm, 1 out of 34 patients (2.9%) did not fulfill Canadian consensus criteria for ME/CFS at baseline, while at six years 16 patients (47.1%) did not fulfill the criteria. Among the RituxME participants, the mean DSQ-SF at six years was 54.0 in the rituximab group and 60.1 in the placebo group; 24 patients (41.5%) in the rituximab group and 17 patients (31.5%) in the placebo group did not fulfil the Canadian criteria at 6 years according to the DSQ-SF algorithm (no comparison with baseline is possible). Table 3 shows the mean DSQ score and specific symptoms present in patients who did, or did not, comply with the DSQ-SF algorithm for defining Canadian consensus criteria at six years.

**Table 3:** DePaul symptom questionnaire–short form. Symptom scores in patients who did or did not comply with Canadian Criteria after six years.

	CycloME		RituxME			
	Cyclophosphamide iv		Rituximab iv		Placebo iv	
	CCC+^1^	CCC-^2^	CCC+	CCC-	CCC+	CCC-
N (%)	18 (52.9)	16 (47.1)	34 (58.6)	24 (41.4)	37 (68.5)	17 (31.5)
DSQ-SF score^3^, mean (SD)	66.4 (13.1)	33.3 (17.4)	68 (12.5)	33.8 (17.4)	69.6 (14.6)	39.2 (15.3)
Fatigue, N (%)^4^	18 (100)	8 (50)	34 (100)	12 (50)	37 (100)	10 (58.8)
PEM, N (%)^4^	18 (100)	7 (43.8)	34 (100)	11 (45.8)	37 (100)	10 (58.8)
Sleep, N (%) ^4^	18 (100)	7 (43.8)	34 (100)	10 (41.7)	37 (100)	8 (47.1)
Neuro/cognitive, N (%)	18 (100)	9 (56.3)	34 (100)	10 (41.7)	37 (100)	4 (23.5)
Pain, N (%)	16 (88.9)	4 (25)	29 (85.3)	11 (45.8)	29 (78.4)	9 (52.9)
Autonomous, N (%)	14 (77.8)	5 (31.3)	25 (73.5)	5 (20.8)	20 (54.1)	4 (23.5)
Neuroendocrine, N (%)	14 (77.8)	6 (37.5)	28 (82.4)	8 (33.3)	32 (86.5)	5 (29.4)
Immune, N (%)	13 (77.2)	3 (18.8)	27 (79.4)	2 (8.3)	27 (73)	2 (11.8)

Abbreviations: CCC = Canadian Consensus Criteria. DSQ-SF = DePaul Symptom Questionnaire, Short Form. PEM = Post-Exertional Malaise. CCC+: Patients who complied with the CCC according to the DSQ-SF algorithm at six-year follow up. CCC-: Patients who did not comply with the CCC according to the DSQ-SF algorithm at six-year follow up. ^3^: Score 0–112, higher score indicates greater symptom burden.

^4^: Patients who rate 2 or higher for both severity and frequency on a 5-point Likert scale (i.e. moderate symptoms or worse, half the time or more) are considered to have a symptom.

### Function level and study specific questionnaire

Mean Function level (%) at baseline was 17.7 in the CycloME trial, 18.8 in the RituxME rituximab- and 17.9 in the RituxME placebo group. At six years, participants from the three groups recorded mean function levels of 38.4, 30.9, and 28.6 respectively (Fig 2, panels C and D).

Patients reported their overall health status at six years compared to the end of the clinical trials at 18/24 months. A healthier status was reported by 16 (47%), 21 (39%), and 23 (40%) patients in the cyclophosphamide, rituximab and placebo groups, respectively. No change was reported by 7 (21%), 16 (30%) and 17 (30%), and overall worsening compared to the end of study by 11 (32%), 17 (32%) and 17 (30%) patients, respectively.

Out of 22 responders at 18 months in the CycloME trial, 20 participated in the six-year follow-up, and SF-36 PF indicated further improvement at six years for 14 patients. Worsening during this period was reported by 6 patients; however, these still had higher SF-36 PF scores at six years than at baseline.

As stated in the original protocols for the two studies, none of the patients received any specialized medical care except the allocated trial intervention during the follow-up periods of 18/24 months. After the end of the trials, 25% of the cyclophosphamide-, 54% of the rituximab-, and 39% of the placebo-treated patients had tried other forms of treatment aimed at their ME/CFS symptoms. Medical treatment with low dose naltrexone was the most frequently attempted treatment. Dietary supplements and vitamins were also common; 14 patients from the CycloME trial and 49 patients from the RituxME trial had performed some dietary adjustments such as a low-carbohydrate, low-FODMAP, gluten-free or other diets, without reporting any systematic changes of symptoms or function level.

In the CycloME trial, six out of 40 patients (15.0%) worked part-time and none fulltime, at baseline. At six years, six out of 34 patients worked part-time and four worked full-time (29.4% in total). Four of the part-time workers and two of the full-time workers at six years did not work at all at baseline. In the RituxME trial, out of 151 patients at baseline, four worked part-time, one full-time and three were students (5.3%). At six years, out of 112 participants, one worked part-time, three worked full-time, and two were students (4.5%).

### Adverse events

More than half the patients in both studies had been diagnosed with one or more new comorbidities or received new diagnoses for pre-existing symptoms/comorbidity at six-year follow-up. There was a broad spectrum of diagnoses, many of which are commonly found in ME/CFS patients, such as irritable bowel syndrome (IBS) and postural tachycardia syndrome (POTS). As such, it is difficult to make assessments on possible associations with study participation. The new diagnoses reported were more or less equally divided between the rituximab and placebo groups in the RituxME trial, and these were also similar in the CycloME trial. Two patients who were treated with rituximab reported symptom exacerbation (pain, muscle spasms) after trial participation, which they perceived to be related to the rituximab treatment. However, there were more patients in the placebo group reporting symptom worsening after end of the trial (Fig 4B). Regarding neoplastic diseases, one patient in the rituximab and one in the placebo group reported diagnoses of breast cancer, and one patient in the cyclophosphamide group had a resection of a benign olfactory meningioma.

In the CycloME trial, five women aged 42 to 51 years at inclusion in the study entered menopause during or after the 18-month study period. One young woman with possible premature menopause reported in the CycloME trial [14] has later given birth to a healthy child and is presently expecting her second, both conceptions without the aid of assisted reproductive technology. Four additional patients (one of whom used hormonal contraceptives) had periods with irregular menstrual bleedings between the end of study and the six-year follow-up.

## Discussion

This study provides six-year follow-up data for patients with ME/CFS enrolled in the two clinical intervention trials RituxME and CycloME. The studies were initiated based on the hypothesis that development of ME/CFS may be associated with an autoimmune pathomechanism with a role for B cells/plasma cells and autoantibodies. RituxME was conducted as a double-blind trial, but after 24 months, the patients were informed of their intervention group allocation, i.e. rituximab or placebo. CycloME was an open-label trial, so the patients had knowledge of their active treatment for all six years.

Patients were included in the two trials in overlapping time-periods with similar inclusion criteria, except that patients with mild ME/CFS were not included in CycloME, and patients with disease duration > 15 years were not included in RituxME. The baseline characteristics were similar in the two trials. The participation rate for this six-year follow-up study was higher for the CycloME trial (94.4%) versus the RituxME trial (74.7%). For the RituxME trial, the participation rate was higher among patients with high symptom burden at baseline. The improvements in mean outcome measures from baseline to 18 months were comparable for participants and non-participants. This does not indicate a likely bias of lower participation rate for patients with worse outcome during the trial, i.e. the data probably do not overestimate the clinical improvements reported.

The six-year follow-up data show that for patients in the CycloME trial, the mean values for SF-36 Physical Function (PF), DSQ-SF and Function level (%) improved during the trial and continued to improve slightly from the end of study to the six-year follow-up. Average scores for SF-36 PF and Function level in patients participating in RituxME remained relatively stable from the end of study to six years.

All PROMs used in the present six-year follow-up indicate greater and more lasting improvements in ME/CFS patients treated with cyclophosphamide compared to participants in the RituxME trial. At six years, patients in the CycloME and RituxME trials reported average SF-36 PF scores of 56.7 and 44.3 respectively. Average age in the CycloME and RituxME study at six years was 47.4 and 44.2 years, while the mean SF-36 PF score in the normal Norwegian population age 40–49 is reported to be 90.7 (SD 16.8) for women and 92.8 (SD 12.6) for men [26]. At six-year follow-up, 44.1% of the CycloME patients had an SF-36 PF of 70 or more. The percentage of patients with SF-36 PF in the normal range (at least 90) was higher, and the percentage of patients with reduction of SF-36 PF of at least 20 points lower, for the patients treated with cyclophosphamide, as compared to patients treated with rituximab or placebo. Long-lasting improvements after cyclophosphamide were also seen for the two other outcomes, Function level and the DSQ-SF scores, in a subgroup of patients. Even though the DSQ-SF algorithm for defining Canadian consensus criteria is less accurate than a full clinical evaluation, it is interesting that 16 patients (47.1%) in the CycloME trial no longer fulfilled the criteria at six years, compared to one patient at baseline. In the RituxME trial at six years, 23 (40.4%) patients in the rituximab group failed to meet the criteria, and 17 (31.5%) in the placebo group. As shown in Table 3, among the patients who no longer met Canadian consensus criteria at six years, almost half still reported core ME/CFS symptoms such as PEM, cognitive problems, fatigue or sleep disturbances at least to a moderate degree and at least half the time. The presence of residual symptoms is hardly surprising, as the patients reports from partial to full recovery.

Based on the observations from the two separate trials using different immunoregulatory drugs, we compared results from the RituxME and CycloME trials in a post-hoc analysis. With similarity in terms of inclusion and follow-up in both trials, such a comparison may be informative for the generation of future hypotheses and testing of interventions. In GLM repeated measures analyses, adjusted for baseline age, sex, study centre and ME/CFS severity, there were significant differences for courses of SF-36 PF and Function level between the CycloME and RituxME patients, in favor of the CycloME group. Although such comparison between trials should be interpreted with caution, and despite the lack of randomized and placebo-controlled data for cyclophosphamide, we suggest that the ME/CFS symptom improvement after cyclophosphamide intervention is a sign of clinical response to treatment. This supports the hypothesis that a subgroup of ME/CFS patients have a variant of an autoimmune disease mechanism which may be targetable by immunomodulatory drugs.

The pathomechanisms for ME/CFS are not established. We published a model suggesting an aberrant immune response after infection, with a role for B lymphocytes, plasma cells and autoantibodies [27]. The lack of efficacy for rituximab as scheduled in the RituxME study could possibly be caused by autoantibody production from CD20 negative, long-lived mature plasma cells [28].

Cyclophosphamide is a cytotoxic drug with immunomodulatory properties. Infusions at the doses and schedule used in the CycloME trial [14] are associated with moderate depletion of several subsets of lymphocytes (CD4 and CD8 T cells, CD19 B cells and CD56/16 NK cells), and also with modest reductions in serum immunoglobulin levels [29]. The broad effect of cyclophosphamide on the different immune cells makes it difficult to pinpoint an exact mechanism for the observed clinical effect in ME/CFS. However, the cytotoxic effects on proliferating cells make inhibition of activated B cells to plasmablasts one plausible possibility. Some studies report higher frequency of T regulatory cells (T regs) in ME/CFS patients [30–32]. Cyclophosphamide affects T regs more than other T cell subsets, such as T helper cells, due to higher proliferation rate [29]. The down-regulation of T cells, the impact on several subsets of lymphocytes and interactions between immune cells, or a reduction in IgG levels -, including putative autoantibodies, are other possible mechanisms of cyclophosphamide in this disease. A hypothesis suggesting an interplay between T regs and chronic viral infections has also been forwarded [33]. Despite the encouraging results from the CycloME trial patients, cyclophosphamide is not an ideal drug for ME/CFS, and further research should aim to elucidate the pathomechanisms of ME/CFS, and additionally to further develop safe and effective immunomodulatory treatment to target disease mechanisms.

Generally, comparison of data between clinical trials from different countries and hospitals is hampered by the use of different outcome measures and, importantly, inclusion of patient populations with different levels of symptom severity. Even with the RituxME and CycloME trials both conducted by our study groups with clear similarity in inclusion criteria and full data sets available for post-hoc analysis, it is difficult to draw definitive conclusions. Thus, when comparing the long-term outcome of different trials, one should keep in mind the differences in patient selection and ME/CFS severity, as assessed e.g. by baseline SF-36 PF or DSQ-SF.

Due to lack of sufficient longitudinal data on ME/CFS disease course and prognosis, it is difficult to assess whether symptom improvement in a clinical trial can be attributed to the intervention or to other factors. During the trials, placebo mechanisms may contribute, and participants can also be affected by being taken care of in a clinical study (“trial effect”). A modest, but evident rate of recovery in the placebo group indicates a potential for spontaneous improvement, suggesting that ME/CFS is in principle a reversible disease.

There is no standard care program for patients with ME/CFS in the Norwegian public health system, and some patients in our trials may have profited from the systematic follow-up involved in a structured clinical trial, with regular physician and nurse consultations. A recent survey study from Norway with 660 patients concluded that the experienced health benefits of some of the most common interventions used for ME/CFS in both primary and specialist health care services were generally low and, at times, negative [34]. There is a need to improve the regular follow-up system for this patient group in primary health care.

This long-term follow-up study of the RituxME trial serves as a report of any late effects of rituximab on the ME/CFS disease course. There were no significant differences in outcome measures between the rituximab and placebo groups, neither during the trial nor in the time interval from two to six years. The percentages of patients achieving substantial increases in SF-36 PF from baseline were similar in the rituximab and placebo groups. At the six-year follow-up, the percentages of patients reporting worsening with SF-36 PF reductions of at least 10 or 20 points from baseline, were slightly higher in the placebo group than in the rituximab group, but with no significant differences. Thus, there were neither convincing evidence of long-term benefit nor harmful effects of rituximab with the schedule and doses given in the trial.

Importantly, the six-year follow-up study did not uncover any serious long-term toxicity between the end of study and six years’ follow-up in either study. Except for the described fertility issues of cyclophosphamide, the incidence of new comorbidity or new diagnoses for pre-existing comorbidities was comparable in all three treatment groups.

During the last 15 years, there have been several observations in our department of oncology of ME/CFS patients who got cancer and who reported that the cancer drug treatment unexpectedly induced beneficial and long-lasting effects on their ME/CFS symptoms. The CycloME trial follow-up at six years shows sustained, clinically meaningful improvements as described. A significantly larger number of patients improved in the CycloME trial compared to both the placebo and the rituximab groups from the RituxME trial. We suggest that the ME/CFS symptom improvement after cyclophosphamide intervention indicates a beneficial effect from intervention in a subgroup of patients. However, we advise patients and physicians not to use cyclophosphamide for ME/CFS patients outside of clinical trials before further research is available. Rather, these data should encourage efforts to better understand disease mechanisms and to search for targeted and less toxic immune modulatory treatment for this patient group.

## Supporting information

S1 Checklist (PDF)

S1 File Study-specific questionnaire. (PDF)

S2 File Table with SF36 domains (Physical function, bodily pain, general health, vitality, social function, mental health) from baseline, at 18 months and at 6 years. (PDF)

S3 File Clinical data, PROMs data from baseline and follow-up. (XLSX)

S4 File Protocol RituxME trial. (PDF)

S5 File Protocol CycloME trial. (PDF)

S6 File Approval by the regional committees for medical and health research ethics in norway. (PDF)

S7 File SPSS analysis code. (PDF)
