# Supplementary material for: Six-year follow-up of participants in two clinical trials of rituximab or cyclophosphamide in Myalgic Encephalomyelitis/Chronic Fatigue Syndrome
Source: PLoS One. 2024 Jul 23;19(7):e0307484. doi: 10.1371/journal.pone.0307484 (PMC11265720; doi:10.1371/journal.pone.0307484)
Supplement: S1 Checklist — (PDF) [file pone.0307484.s001.pdf]

STROBE Statement—checklist of items that should be included in reports of observational studies

	Item No.	Recommendation	Page No.	Relevant text from manuscript
**Title and abstract**	1	(*a*) Indicate the study's design with a commonly used term in the title or the abstract	1	Six-year follow-up of participants in two clinical trials
		(*b*) Provide in the abstract an informative and balanced summary of what was done and what was found	1-2	
**Introduction**				
Background/rationale	2	Explain the scientific background and rationale for the investigation being reported	3-6	
Objectives	3	State specific objectives, including any prespecified hypotheses	6	we wanted to explore the long-term course of disease symptoms and possible late side effects of treatment. Since the trials were similar in design, especially regarding time of inclusion, inclusion criteria and outcome measures, we wanted to compare long-term outcome in the two trials.
**Methods**				
Study design	4	Present key elements of study design early in the paper	10	In the 6-year follow-up study, we used patient-reported outcome measures (PROMs). We compared values at baseline, at 18 months, and at 6-year follow up for the CycloME trial and RituxME trial (rituximab and placebo groups).
Setting	5	Describe the setting, locations, and relevant dates, including periods of recruitment, exposure, follow-up, and data collection	6-7	The follow-up study for RituxME was performed from

				01.05.2021 to 01.09.2021, 6 years after inclusion. The follow-up study for CycloME was performed from 25.10.2022 to 31.01.2023, 6-7 years after inclusion. The participants received questionnaires by mail and returned them to study-coordinator at Haukeland University Hospital.
Participants	6	(*a*) *Cohort study*—Give the eligibility criteria, and the sources and methods of selection of participants. Describe methods of follow-up<br>*Case-control study*—Give the eligibility criteria, and the sources and methods of case ascertainment and control selection. Give the rationale for the choice of cases and controls<br>*Cross-sectional study*—Give the eligibility criteria, and the sources and methods of selection of participants	6-7	
		(*b*) *Cohort study*—For matched studies, give matching criteria and number of exposed and unexposed<br>*Case-control study*—For matched studies, give matching criteria and the number of controls per case	NA	
Variables	7	Clearly define all outcomes, exposures, predictors, potential confounders, and effect modifiers. Give diagnostic criteria, if applicable	10-11	
Data sources/ measurement	8*	For each variable of interest, give sources of data and details of methods of assessment (measurement). Describe comparability of assessment methods if there is more than one group	10-11	
Bias	9	Describe any efforts to address potential sources of bias	11	For comparison of outcomes (SF-36 PF and Function level) over time (baseline, 18 months, 6 years), by treatment groups (cyclophosphamide, rituximab, placebo), adjustments were made for age, sex and baseline

				ME/CFS severity (included in the model as covariates).
Study size	10	Explain how the study size was arrived at	NA	

Continued on next page

Quantitative variables	11	Explain how quantitative variables were handled in the analyses. If applicable, describe which groupings were chosen and why	NA
Statistical methods	12	(*a*) Describe all statistical methods, including those used to control for confounding	11-12
		(*b*) Describe any methods used to examine subgroups and interactions	11-12
		(*c*) Explain how missing data were addressed	Page 13, Fig 1, Fig 2 Legends
		(*d*) *Cohort study*—If applicable, explain how loss to follow-up was addressed<br>*Case-control study*—If applicable, explain how matching of cases and controls was addressed<br>*Cross-sectional study*—If applicable, describe analytical methods taking account of sampling strategy	
		(*e*) Describe any sensitivity analyses	NA
**Results**			
Participants	13*	(a) Report numbers of individuals at each stage of study—eg numbers potentially eligible, examined for eligibility, confirmed eligible, included in the study, completing follow-up, and analysed	13, Table 1, Fig 1
		(b) Give reasons for non-participation at each stage	Fig 1
		(c) Consider use of a flow diagram	Fig 1
Descriptive data	14*	(a) Give characteristics of study participants (eg demographic, clinical, social) and information on exposures and potential confounders	Table 1, page 13
		(b) Indicate number of participants with missing data for each variable of interest	Table 1, Fig 2 Legend
		(c) *Cohort study*—Summarise follow-up time (eg, average and total amount)	13
Outcome data	15*	*Cohort study*—Report numbers of outcome events or summary measures over time	13-17
		Case-control study—Report numbers in each exposure category, or summary measures of exposure	
		Cross-sectional study—Report numbers of outcome events or summary measures	
Main results	16	(*a*) Give unadjusted estimates and, if applicable, confounder-adjusted estimates and their precision (eg, 95% confidence interval). Make clear which confounders were adjusted for and why they were included	13-17
		(*b*) Report category boundaries when continuous variables were categorized	NA

	(*c*) If relevant, consider translating estimates of relative risk into absolute risk for a meaningful time period	NA

Continued on next page

Other analyses	17	Report other analyses done—eg analyses of subgroups and interactions, and sensitivity analyses	17	
**Discussion**				
Key results	18	Summarise key results with reference to study objectives	20	
Limitations	19	Discuss limitations of the study, taking into account sources of potential bias or imprecision. Discuss both direction and magnitude of any potential bias	22	Generally, comparison of data between clinical trials from different countries and hospitals is hampered by the use of various outcome measures and, importantly, inclusion of patient populations with different levels of symptom severity.
Interpretation	20	Give a cautious overall interpretation of results considering objectives, limitations, multiplicity of analyses, results from similar studies, and other relevant evidence	19-24	
Generalisability	21	Discuss the generalisability (external validity) of the study results	19-24	
**Other information**				
Funding	22	Give the source of funding and the role of the funders for the present study and, if applicable, for the original study on which the present article is based	12	

*Give information separately for cases and controls in case-control studies and, if applicable, for exposed and unexposed groups in cohort and cross-sectional studies.

Note: An Explanation and Elaboration article discusses each checklist item and gives methodological background and published examples of transparent reporting. The STROBE checklist is best used in conjunction with this article (freely available on the Web sites of PLoS Medicine at http://www.plosmedicine.org/, Annals of Internal Medicine at http://www.annals.org/, and Epidemiology at http://www.epidem.com/). Information on the STROBE Initiative is available at www.strobe-statement.org.
