# Supplementary material for: Six-year follow-up of participants in two clinical trials of rituximab or cyclophosphamide in Myalgic Encephalomyelitis/Chronic Fatigue Syndrome
Source: PLoS One. 2024 Jul 23;19(7):e0307484. doi: 10.1371/journal.pone.0307484 (PMC11265720; doi:10.1371/journal.pone.0307484)
Supplement: S1 File — (PDF) [file pone.0307484.s002.pdf]

Follow-up study, CycloME trial

Study ID		Initials	
Date			

In the CycloME trial we used a scale (0 to 100%) for «**function level**», which you recorded every two weeks. You based your answers on a form (attached) with examples of different function levels. 100% corresponds to a completely healthy state.

- You were included in the CycloME trial _*[date]*_______.
  In the beginning of the trial you recorded a function level of _*[xx]*_%.
- You completed follow-up in the trial _ *[date]* _________.
  At the end of the trial you recorded a function level of __*[xx]* _ %.

1. How would you estimate your current function level (on average, over the last three months)?

[ ] % (from 0 to 100%)

2. Compared to the time you completed trial follow-up, how do you feel now?

- ❑ Better
- ❑ Unchanged
- ❑ Worse

3. What is your present employment status?

- ❑ Work assessment allowance/ disability benefit
- ❑ Student
- ❑ Homemaker
- ❑ Retired
- ❑ Unemployed
- ❑ Employed full-time
- ❑ Employed part-time
- ❑ On sick leave
- ❑ Occupational rehabilitation

Følsomhet Intern (gul)

4. Have you been diagnosed with any new or different disease/condition **after** the end of trial?

❑ Yes

If yes: please specify: ____

❑ No

5. Have you experienced worsening or improvement of **other** diseases/conditions **after** the end of trial?

❑ Yes, improvement of ____

❑ Yes, worsening of ____

❑ No

6. Have you received any treatment aimed at your ME/CFS **after** the end of trial? Please specify.

❑ No treatment

❑ Drug treatment:____

❑ Cognitive/other therapy: ____

❑ Graded exercise therapy: ____

❑ Other: ____

7. Have you made any systematic changes to your diet **after** the end of trial? Please specify.

❑ No change

❑ Started low carb diet

❑ Started FODMAP

❑ Started gluten-free diet

❑ Other diet – specify: ____

____

Følsomhet Intern (gul)

- ☐ New dietary supplements: ____

  ____

- ☐ Stopped dietary supplements: ____

  ____

Følsomhet Intern (gul)

8. Have you experienced that any treatment, dietary change or other measures have significantly affected your ME/CFS symptoms and/or activity level over time?

❑

If yes, please specify: ____

❑ No

9. For female participants: Have you experienced any temporary or lasting changes in your menstrual cycle during or after the CycloME trial?

❑ Yes

If yes, please specify:

What kind of change? ____

When did the changes occur? ____

Have the changes persisted until the present time? _____________________

❑ No

❑ Don't know/not applicable

10. Comments or additional information:

____

____

____

____

____

____

____

____

____

____

____

____

Thank you very much for your help!

Følsomhet Intern (gul)
