# Supplementary material for: Six-year follow-up of participants in two clinical trials of rituximab or cyclophosphamide in Myalgic Encephalomyelitis/Chronic Fatigue Syndrome
Source: PLoS One. 2024 Jul 23;19(7):e0307484. doi: 10.1371/journal.pone.0307484 (PMC11265720; doi:10.1371/journal.pone.0307484)
Supplement: S2 File — (PDF) [file pone.0307484.s003.pdf]

S2 File. SF36 domains (Physical Function, Bodily pain, General health, Vitality, Social Function, Mental Health) from baseline, at 18 months and at 6 years

Trial	CycloME			RituxME					
Drug	Cyclophosphamide i.v. (n=34)			Rituximab iv. (n=58)			Placebo i.v. (n=54)		
	Baseline	18 months	6 years	Baseline	18 months	6 years	Baseline	18 months	6 years
PF[1] Mean (SD)	35.4 (17.4)	54.4 (27.4)	56.7 (27.6)	32.9 (22.3)	42.4 (25.0)	45.5 (29.5)	32.3 (16.9)	46.3 (25.6)	43.1 (28.6)
BP[2] Mean (SD)	34.5 (21.3)	45.9 (25.9)	47.3 (25.5)	35.1 (20.6)	45.6 (24.9)	47.5 (24.8)	36.4 (20.2)	50.3 (25.0)	45.9 (22.7)
GH[3] Mean (SD)	31.4 (12.2)	38.1 (18.8)	39.2 (23.4)	28.6 (13.3)	32.2 (17.1)	36.6 (23.1)	28.1 (13.3)	32.5 (18.3)	33.4 (18.5)
VT[4] Mean (SD)	20.3 (11.6)	37.4 (23.0)	35.6 (23.5)	21.3 (14.4)	31.2 (21.1)	32.2 (21.8)	21.0 (14.2)	32.5 (23.1)	29.8 (21.4)
SF[5] Mean (SD)	17.6 (15.1)	38.2 (34.0)	39.7 (31.9)	19.2 (19.9)	32.1 (28.1)	36.0 (31.7)	19.4 (21.1)	37.7 (30.1)	34.3 (30.5)
MH[6] Mean (SD)	76.6 (13.7)	78.7 (14.1)	76.6 (15.1)	73.1 (16.0)	75.7 (15.6)	73.5 (16.1)	75.5 (18.1)	72.5 (20.4)	73.0 (20.7)

[1]PF; Physical Function, [2]BP; Bodily Pain, [3]GH; General Health, [4]VT; Vitality, [5]SF; Social Function, [6]MH Mental Health

Følsomhet Intern (gul)
