# Supplementary material for: Six-year follow-up of participants in two clinical trials of rituximab or cyclophosphamide in Myalgic Encephalomyelitis/Chronic Fatigue Syndrome
Source: PLoS One. 2024 Jul 23;19(7):e0307484. doi: 10.1371/journal.pone.0307484 (PMC11265720; doi:10.1371/journal.pone.0307484)
Supplement: S5 File — (PDF) [file pone.0307484.s006.pdf]

CYCLOPHOSPHAMIDE IN MYALGIC ENCEPHALOPATHY / CHRONIC FATIGUE SYNDROME

Part A, for up to 40 patients suffering from ME/CFS: An open phase II study with 6 infusions of cyclophosphamide at 4 week intervals and 18 months follow-up.

(Part B, for up to 20 patients suffering from severe or very severe ME/CFS: An exploratory study with up to 6 infusions of cyclophosphamide at 4 week intervals in collaboration with local health services.)

Protocol code: KTS-7-2015

EudraCT: 2014-004029-41

ClinicalTrials.gov: NCT02444091

Version	Date	Approved	Description of alterations
1.0	22.09.2014	07.11.14 (EC)	N/A
2.0	08.12.2014	09.11.14 (NoMA)	Changes to exclusion criteria and sample size, replacement of «drop-outs», part A: project plan and collaboration, laboratory analyses at baseline. Added details on: part A: inclusion criteria for ergospirometry, part B: cut-off value for neutrophilic granulocytes, cryopreservation, use of other medications, data handling and archives,
2.1	18.02.2015	Submitted for information only.	Adjustments to list of staff members and investigators. Response for part A endpoints will be reported separately for naïve ME/CFS patients (not previously treated with rituximab), for non-responders after previous rituximab intervention, for responders after rituximab with subsequent relapse, and for all included patients as a whole. Details on use of other medications for part B.
3.0	01.08.16	13.10.16	Extended follow-up to 18 months.

Signatures (place, date, name)

____________________ ____________________ ____________________

____________________ ____________________ ____________________

____________________ ____________________ ____________________

TRIAL SITES AND RESPONSIBLE STAFF MEMBERS 4
Study management 4
Study coordinator 5
Safety board 5
Immunological analyses 5
Biobank 5
Study monitoring 6
Cyclophosphamide intervention 6
Sensewear armband for activity registration 6
Endothelial function by Flow-Mediated Dilation (FMD) 7
Microvascular endothelial function by Post Occlusive Reactive Hyperemia (PORH) 7
Ergospirometry 7

BACKGROUND AND PROJECT DESCRIPTION 7
Published pilot study 8
Published double-blind, randomized and placebo controlled study 8
Open phase II studies of Rituximab in ME/CFS 8
New randomized phase III study in progress (RituxME) 9
Hypothesis 10
Pilot observations of ME/CFS patients with breast cancer 12
Pilot treatment with cyclophosphamide in ME/CFS at HUS 12

NEW OPEN PHASE II STUDY: CYCLOPHOSPHAMIDE IN MYALGIC ENCEPHALOPATHY / CHRONIC FATIGUE SYNDROME 15

PART A 15
PROJECT PLAN, ORGANISATION AND COLLABORATION, PART A 15
THE MAIN STUDY OBJECTIVE, PART A 16
ENDPOINTS 16
Primary endpoint, part A 16
Secondary endpoints, part A 17
DESIGN, PART A 18
PATIENT SAMPLE, PART A 18
INCLUSION CRITERIA, PART A 19
EXCLUSION CRITERIA, PART A 19
CONSULTATION, PART A 19
INTERVENTION, PART A 19
DATA COLLECTION AND DATA MANAGEMENT, PART A 20
Modified DePaul and HADS questionnaires 21
Self-reported symptom score 22
Self-reported symptom score before intervention 22
Self-reported symptom change during follow-up 22
”Total function level" 23
SF-36 questionnaire on health, and analysis 23
Fatigue Severity Scale 23
Doctor’s registration at baseline and follow-up 24
Sensewear armbands for recording activity level at home 24
EXAMINATIONS AND REGISTRATION BEFORE INTERVENTION, PART A 25
Clinical assessment 25
LABORATORY TESTS AT BASELINE, PART A 26
Routine laboratory tests 26
Immunology 26
Endocrinology 26

Microbiology 26
Biobank blood samples 26
SENSEWEAR ARMBAND FOR ACTIVITY REGISTRATION AT BASELINE, PART A 26
ENDOTHELIAL FUNCTION (FMD) AND MICROVASCULAR FUNCTION AT BASELINE, PART A 27
ERGOSPIROMETRY AT BASELINE, PART A 27
CYCLOPHOSPHAMIDE INFUSIONS EVERY FOUR WEEKS, PART A 27
EXAMINATIONS AND REGISTRATION AT 3, 6, 9, 12, 15 AND 18 MONTHS, PART A 27
Clinical assessment with registration and entry in medical records 27
Laboratory tests 28
Blood samples for biobank 28
SENSEWEAR ARMBAND FOR ACTIVITY REGISTRATION AT 7-9, 11-12 AND 17-18 MONTHS 28
ENDOTHELIAL/MICROVASCULAR FUNCTION TESTS AT 7-9 AND/OR 11-12 MONTHS 28
ERGOSPIROMETRY AT 7-9, ALTERNATIVELY AT 11-12 MONTHS 28
VISIT AFTER 12 MONTHS, AND FINAL VISIT AFTER 18 MONTHS. 28

FOR BOTH PARTS A AND B OF THE STUDY: 30
MONITORING 30
BIOBANK FOR BIOLOGICAL STUDIES AND IMMUNOPHENOTYPING 30
PATIENT WITHDRAWAL DURING STUDY 31
ADVERSE EVENTS, SAFETY BOARD, SIDE EFFECTS 31
Safety board and safety profile 32
Side effects 32
ETHICAL CONSIDERATIONS 35
FUNDING 36
PUBLICATION 37
APPLICATIONS FOR APPROVAL 37

REFERENCES 37

APPENDICES

A. Canadian criteria for ME/CFS.
B. Modified DePaul questionnaire – baseline (part A).
C1. Patient instructions on self-reporting (part A)
C2. Patient instructions on self-reporting (part B)
D1. Self-report form for ME/CFS symptoms before intervention (part A)
D2. Self-report form for ME/CFS symptoms before intervention (part B)
E1. Self-report form for ME/CFS symptom change every second week (part A)
E2. Self-report form for ME/CFS symptom change every second week (part B)
F1. SF-36 (v1.2) health questionnaire (parts A and B)
F2. Fatigue Severity Scale (FSS) questionnaire (part A)
F3. Hospital Anxiety and Depression Scale (HADS) questionnaire (part A)
G1. Form for doctor's registration of ME/CFS at baseline and follow-up (part A)
G2. Form for doctor's registration of ME/CFS at baseline and follow-up (part B)
H1. Endothelial function testing (parts A and B)
H2. Ergospirometry, two consecutive days (part A)
I. Blood samples for biobank and immunophenotyping (parts A and B).
J1. Patient information and consent form for clinical study (part A)
J2. Patient information and consent form for clinical study (part B)
K. Flow charts, part A
L. Serious Adverse Events and CIOMS report forms (parts A and B)

TRIAL SITES AND RESPONSIBLE STAFF MEMBERS

Study management

The study is managed from the Dept. of Oncology and Medical Physics at Haukeland University Hospital (HUS), by Head of Dept., Professor Olav Mella and Senior Consultant Øystein Fluge.

Sponsor's representative/PI

Professor/Head of Dept. Olav Mella, MD, PhD
Dept. of Oncology and Medical Physics,
Haukeland University Hospital, 5021 Bergen
Tel: +47 55 97 20 69
Mobile: +47 90 99 01 85
E-mail: olav.mella@helse-bergen.no
E-mail: olav_mella@hotmail.com

Project manager/Investigator

Senior Consultant Øystein Fluge, MD, PhD
Dept. of Oncology and Medical Physics,
Haukeland University Hospital, 5021 Bergen
Tel.: +47 55 97 20 10
Mobile: +47 93 04 40 24
E-mail: oystein.fluge@helse-bergen.no
E-mail: oystein.fluge@gmail.com

Investigator

Dr. Ingrid Gurvin Rekeland
Dept. of Oncology and Medical Physics,
Haukeland University Hospital, 5021 Bergen
Tel.: +47 55 97 20 10
Mobile: +47 99 00 56 97
E-mail: ingridgurvin@hotmail.com
E-mail: ingrid.gurvin.rekeland@helse-bergen.no

Investigator

Dr. Irini Ktoridou-Valen
Dept. of Oncology and Medical Physics,
Haukeland University Hospital, 5021 Bergen
Tel.: +47 55 97 20 10
Mobile: +47 46 66 49 28
E-mail: eikt@helse-bergen.no

Investigator

Alexander Fosså
Dept. of Oncology
The Norwegian Radium Hospital, Oslo University Hospital
Ullernchausseen 70

0379 Oslo
Tel: +47 22 93 40 00
E-mail: aff@ous-hf.no

Study coordinator

Study nurse Kari Sørland
Dept. of Oncology and Medical Physics,
Haukeland University Hospital, 5021 Bergen
Tel: +47 55 97 04 39
Mobile: +47 47 71 93 98
E-mail: kari.sorland@helse-bergen.no
E-mail: karisorland@hotmail.com

In collaboration with:
In collaboration with:
The Clinical Trial Unit, Haukeland University Hospital
Mari Holsen, study nurse and Marianne Lehmann, study nurse
Tel.: +47 55972890
E-mail: mahh@helse-bergen.no
E-mail: marianne.emblem.lehmann@helse-bergen.no

Safety board

Olav Dahl, Professor, Senior Consultant, Dept. of Oncology and Medical Physics, Haukeland University Hospital (Chairman)
Ola Didrik Saugstad, Professor, Senior Consultant, Institute of Paediatrics, University of Oslo
Tel. (Olav Dahl): +47 55972018
Mobile: +47 91884617
E-mail: olav.dahl@helse-bergen.no

Immunological analyses

Head of Dept. Einar K. Kristoffersen, Professor
Dept. of Transfusion Medicine and Immunology
Haukeland University Hospital, 5021 Bergen
Tel.: +47 55974683
E-mail: einar.kleboe.kristoffersen@helse-bergen.no

Biobank

Ove Bruland, PhD
Centre for Medical Genetics and Molecular Medicine
Haukeland University Hospital
5021 Bergen
Tel.: +47 55975324
E-mail: ove.bruland@helse-bergen.no

Kine Alme, M.Sc.

Dept. of Oncology and Medical Physics,
Haukeland University Hospital
5021 Bergen
Tel.: +47 55976255
E-mail: kine.alme@helse-bergen.no

Kristin Risa, Cand. Scient
Dept. of Oncology and Medical Physics,
Haukeland University Hospital
5021 Bergen
Tel.: +47 55976255
E-mail: kristin.risa@helse-bergen.no

Study monitoring

Clinical monitor Ingunn H. Anundskås
Dept. of Research and Development,
The Patient Safety Unit
Haukeland University Hospital
5021 Bergen
Tel: +47 92 86 01 78
E-mail: ingunn.heie.anundskaas@helse-bergen.no

Cyclophosphamide intervention

Part A: Infusions are administered at the Clinical Trial Unit, Haukeland University Hospital.

Patients living in East Norway who are included in part A may receive cyclophosphamide infusions no. 2, 3, 5 and 6 at the chemotherapy unit at the Norwegian Radium Hospital under the supervision of Senior Consultant Alexander Fosså, MD.

Part B: Treatment and follow-up of patients will be performed by the patients' local health services in close collaboration with the study management at HUS.

Sensewear armband for activity registration

Exercise therapist Tor Helge Wiestad and study nurse Anne Falch.
The Cancer Centre for Education and Rehabilitation
Dept. of Oncology and Medical Physics,
Haukeland University Hospital
5021 Bergen
Tel.: +47 55 97 20 93
E-mail: anne.falch@helse-bergen.no
E-mail: tor-helge.wiestad@helse-bergen.no

Endothelial function by Flow-Mediated Dilation (FMD)

Analysis are performed by study nurse Kari Sørland under the supervision of:
Miriam K. Sandvik, MD, PhD and Cardiologist Elisabeth Leirgul, MD, PhD
Haukeland University Hospital, 5021 Bergen.
E-mail: miriamsandvik@gmail.com elisabeth.leirgul@gmail.com

Microvascular endothelial function by Post Occlusive Reactive Hyperemia (PORH)

Analysis are performed by study nurse Kari Sørland.

Ergospirometry

Exercise therapists Espen K. Krohn-Hansen, Renate Jørgensen and Tor Helge Wiestad.
The Cancer Centre for Education and Rehabilitation
Dept. of Oncology and Medical Physics,
Haukeland University Hospital
5021 Bergen
E-mail: tor-helge.wiestad@helse-bergen.no

BACKGROUND AND PROJECT DESCRIPTION

Myalgic Encephalomyelitis/Chronic Fatigue Syndrome (ME/CFS) is characterised by pathological exhaustion and malaise, particularly after strenuous activity, in conjugation with cognitive symptoms such as difficulties with concentration and memory, sensory hypersensitivity, pain (typically muscle and joint pain and new-onset headaches), sleep disturbances as well as a variety of symptoms from the autonomous nervous system. ME/CFS affects approximately 0.1-0.2 % using strict diagnostic criteria [1], and must be differentiated from more general fatigue, which affects a larger percentage of the population. Patients with serious ME/CFS have considerably reduced quality of life, and the condition carries great public socio-economic costs. In recent years, several findings have been published supporting immune dysregulation [2,3], abnormalities in the composition of cerebrospinal fluid [4], reduced cerebral circulation [5] and changes in EEG [6]. An epidemiological study has shown that elderly ME/CFS patients are at an increased risk of developing B-cell-derived non-Hodgkin lymphoma. This susceptibility to B-cell-derived lymphoma has also been observed in other conditions where chronic immune activation is the recognized pathological process. The cause of ME/CFS is unknown, and no universally accepted and effective treatment exists. A lack of reliable biomarkers means the diagnosis is mainly based on the patient’s own experience of the illness. Diagnostic criteria are used to separate the ME/CFS patients from other conditions characterized by general fatigue [8].

At the Oncology Dept. at Haukeland University Hospital a patient observation was recorded. The patient was diagnosed with Hodgkin’s lymphoma, but also had a 7 year long history of stable and debilitating ME/CFS She experienced a recurrence of her HL after her primary treatment, and after several chemotherapy regimens including high-dose treatment with autologous stem cell transplant, she has now been recurrence-free for 7 years. During one of the chemotherapy treatment regimens (MIME), she experienced a significant improvement in all ME/CFS related symptoms, which started 6-7 weeks after commencing chemotherapy. Her ME/CFS symptoms gradually relapsed after 5 months of remission.

Published pilot study

The above-mentioned patient and another two pilot patients with ME/CFS were treated with the monoclonal anti-CD20 antibody rituximab (Mabthera®), which depletes B-lymphocytes effectively and selectively. All three pilot patients had significant but limited duration responses affecting their entire ME/CFS symptomatology [9]. The first two pilot patients had an "early response pattern", showing improvement from approx. 6-7 weeks after infusion, with response duration of 3-4 months. The third patient had a "late response pattern", showing significant improvement from approx. 22 weeks after infusion and a similar response duration of 4 months followed by gradual relapse. Subsequent experience has shown that the "late" response pattern is the most common pattern.

Published double-blind, randomized and placebo controlled study

The Oncology Dept. at Haukeland University Hospital has, in collaboration with the Dept. of Neurology, conducted a double-blind and placebo controlled study with 30 patients. Specifically, half of the patients were given two infusions of rituximab 500mg/ m^2 with two weeks' interval, and the other half were given the equivalent two infusions with saline solution. Patients were followed for 12 months (KTS-1-2008). The study was published in PLoS One [10].

Overall response was defined from the self-reported Fatigue score. Statistical analysis for repeated measurements of Fatigue score showed a significant interaction between time after treatment and intervention group (p=0.018). In other words, the Fatigue score development was significantly different in favour of the rituximab group. The differences between the groups were most evident from 6-10 months after intervention, correlating with the secondary endpoint. The primary end point, which was predefined as 3 months after intervention, was negative.

Overall response, defined (exploratively and post-hoc) as significant and lasting improvement in Fatigue score was registered in 10 patients in the rituximab group (67%, 95% CI 41%-85%) and in only 2 patients in the placebo group (13%, 95% CI 4%-38%) (p=0.003). The average response duration within the 12 months follow-up period for the 10 responders in the rituximab group was 25 weeks (duration 8-44). Four patients had response durations beyond the study period (12 months), and after more than 4 years two patients are still in complete remission.

Open phase II studies of Rituximab in ME/CFS

The open phase II study (KTS-2-1010, no placebo group) was brought to a conclusion in February 2014. The study investigated the dose-response relationship of Rituximab in ME/CFS treatment. Recruiting was completed in February 2011with 29 included patients (including two pilot patients). Rituximab was administered as two IV infusions with two weeks' interval (corresponding to the randomized study), followed by maintenance infusions of Rituximab after 3, 6, 10 and 15 months. All patients have now completed a minimum of 36 months follow-up, and approx. 70 % have experienced clinical improvement (response) according to pre-defined criteria. A major response was detected in 14 patients, a moderate response in four patients, and a "marginal" response in three patients. These responses were defined as major, moderate or marginal based on the self-reported symptoms recorded fortnightly during follow-up, changes in the quality of life questionnaire SF-36, the patients' own perception and the doctors' clinical assessment. The patients with "marginal" response

met predefined response criteria, but the response duration was short and occurred late in the follow-up period, and is presumed to be unrelated to the intervention. Seven patients (25 %) experienced no response.

In the 14 patients with major response, we have seen a considerable change in the pattern of symptoms, usually involving an improvement in all ME/CFS-related symptoms, and with an average response duration of 108 weeks (during the study period of 36 months). In the four patients with moderate response, the average response duration was 68 weeks. At study conclusion (36 months), 12 out of 21 responders showed continued response, whilst the remaining 9 have experienced various degrees of relapse during the last year. . It seems clear that maintenance treatment with repeated infusions of Rituximab cause a significantly increased response duration compared to the observed response duration after two Rituximab infusions alone [10].
Nevertheless, per February 2014 approx. one third of the patients are either non-responders or have recorded marginal "response" after Rituximab maintenance treatment. In addition, approx. half of the major and moderate responders suffer various degrees of relapse after 36 months follow-up.

Results from the open phase II study were published in July 2015.

An open phase II study with Rituximab induction and maintenance treatment as described above is also being conducted with up to 15 patients with very severe ME/CFS (KTS-3-2010). By September 2014 eight patients were included in this study, four of which have been almost constantly bedridden for years. We have experienced great logistical challenges transporting these very ill patients to a hospital, and find it difficult in a busy oncology ward to provide the level of seclusion required. Moderate response is recorded in one of the eight patients, while two (very severely ill) have experienced a beneficial effect on the symptoms without satisfying the response criteria. Our impression based on these dates is that patients with very severe illness are less likely to respond to B-cell depletion using Rituximab.

New randomized phase III study in progress (RituxME)

The published study in Plos One [10] has limitations. It was explorative in nature, being the first study to examine B-cell depletion as a principle of treatment in ME/CFS. The endpoint for expected response in the protocol turned out to be too early and the study was small. In order to confirm or disprove the association of B-cell depletion with statically and clinically significant responses in ME/CFS the study must therefore be repeated in larger patient groups, with a more optimal Rituximab dosage interval and predefined end points based on our more recent experience.

The new, randomized, double-blind and placebo controlled national multi-centre trial RituxME commenced in September 2014 at five study centres: Haukeland University Hospital, St. Olav's Hospital (Trondheim), Oslo University Hospital, Notodden Hospital and the University Hospital of North Norway (Tromsø). The trial is part funded by the Research Council of Norway, the Ministry of Health and Care Services, the MEandYou Foundation and the Norwegian ME Association. 152 included patients will receive two infusions two weeks apart of rituximab (500 mg/m^2, max 1000 mg) or placebo, followed by maintenance infusjons of rituximab (500 mg, fixed dose) or placebo after 3, 6, 9 and 12 months. The trial will remain double-blind until the last patient has completed 24 months follow-up.

Hypothesis

Our hypothesis is that ME/CFS is caused by a form of immune system dysregulation, often triggered after infections. The mechanism could be a type of autoimmune or autoinflammatory process. This assumption is based on the course of response and relapse of ME/CFS symptoms following B-cell depletion. While the B-cells are reduced to very low levels in peripheral blood within days or weeks after commencing Rituximab infusions, there is a «delay» of 2 to 11 months before initial clinical responses are reported. We propose that this delayed response pattern could correlate with a gradual elimination of (auto)antibodies. The response rates as well as the course of response and relapse are consistent with observations following Rituximab treatment in auto-immune conditions such as rheumatoid arthritis. The overrepresentation of women, a proven genetic predisposition [11], and the occurrence of other autoimmune illnesses in the family of ME/CFS patients are other factors that all suggest a possible immunological pathogenesis. A proven increased risk of B-cell lymphoma in elderly ME/CFS patients [7] also suggests that the patients have a chronically activated B-cell system.

This is an evolving hypothesis, and there is ongoing laboratory work being carried out in order to investigate the aetiology and pathogenesis of this disease.
B-cell depletion is an intervention that causes significant disruption to the coordinated action of the immune system, and several other possible interpretations to our findings could therefore be relevant. The effect could be related to reinforcement of Th1-responses after Rituximab treatment and reduction of active B-cells, or due to non-B cell mediated effects such as an impact upon T-cell antigen presentation, or on the regulation of other effector cells in the innate immune system such as monocytes/macrophages or dendritic cells [12] . It is also possible that the effect is related to the elimination of B-lymphotrope viruses such as the Epstein Barr virus (EBV) caused by B-cell depletion.
B-cell depletion in ME/CFS patients has resulted in varying responses, from no response (1/3), via moderate response, to major response. Some patients experience significant changes, elimination of all symptoms and a sense of full recovery. Generally, we have observed that all ME/CFS related symptoms are affected during response, including fatigue related symptoms, cognitive symptoms, pain and ”other symptoms”. This indicates that treatment with Rituximab affecting a central pathogenesis either directly or indirectly.

The determination of a target for this type of immune-mediated process will be crucial to the understanding of the ME/CFS pathogenesis. Furthermore the identification of the target could be used as a starting point for the identification of a specific biomarker, and may open up novel therapeutic targets for effective symptomatic treatment, which interferes directly with the effector system for symptom maintenance.
A study measuring reactive vasodilation of arteria brachialis after 4-5 min. occlusion using a blood pressure cuff (flow mediated vasodilation, FMD), concludes that ME/CFS-patients have endothelial dysfunction [13]. In collaboration with the Department of Cardiology at HUS, we have measured endothelial function using FMD in a total of 16 ME/CFS patients. Average FMD was 3.5%, and five patients had an FMD < 1%. This is in comparison to an average FMD of 8.5% measured in healthy women, using the same equipment and protocol, by the same two doctors. Only one out of 66 healthy women had an FMD < 2%. Thus our preliminary data support the findings in the above-mentioned study [13].
Endothelial dysfunction is a risk factor for cardiovascular diseases [14], and a slight to moderate reduction in FMD is also associated with autoimmune systemic diseases [15]. A

slight or moderate association between FMD and depression is also described in some studies [16]. Thus the preliminary data from our analyses in ME/CFS patients show a significantly reduced FMD, which could be an essential discovery with implications for the pattern of symptoms found in this disease.

A main attribute of endothelial dysfunction measured with FMD is an inadequate nitrogen monoxide (NO) synthesis in endothelial cells. Based on knowledge of several functions of NO we propose that a relative lack of NO could contribute to the ME/CFS symptoms. NO causes vasodilatation, and is an important factor in the autoregulation of blood flow, where "shear stress" in the vessel wall constitutes an important signal for eNOS activation. The enzyme eNOS is a catalyst for the production of NO in endothelial cells. Amongst the many functions of NO that appear to be of relevance to ME/CFS symptoms NO is a neural transmitter that can affect memory and concentration. In addition, NO relaxes smooth muscle cells and changes the motility in the gastrointestinal tract and the urogenital system, and NO affects platelet aggregation and the contractility of the heart [17]. NO also affects the immune system, where iNOS regulates the activity of macrophages, T-lymphocytes, antigen-presenting cells, mast cells, neutrophil granulocytes and NK cells [18]. Low levels of NO cause sensory hyperexcitability, particularly affecting Kv channels (voltage-gated potassium channels), in which an increase in NO provides control over neuronal excitability [19,20]. ”Spillover" from the various sources of nitrite (endothelial cell eNOS, neuron nNOS, immune cell iNOS contribute to the total level of NO, in a complex interplay, which is very hard to regulate with drug interventions.

A number of data from the literature relating to ME/CFS can be explained by a hypothesis that involves a dysregulated NO system as a contributory effector system for symptom maintenance. Several studies have shown increased lactate levels in cerebrospinal fluid in patients with ME/CFS[21,22]. Furthermore, a local increase in lactate levels has been detected in cerebral tissue of ME/CFS patients after mental and physical strain, using MR spectroscopy amongst other techniques. Repeated stress tests on two consecutive days show that ME/CFS patients reach anaerobic threshold at a lower level of exercise and importantly at a low oxygen uptake. The patients have lower scores on day 2, which are also associated with an increased lactate production from anaerobic glycolysis and reduced mitochondrial ATP production [23]. A recently published study shows that reduced oxygen extraction from blood in ME/CFS patients and a concomitant reduction in oxygen uptake in muscle cells and probably other tissue as well [24]. Reduced NK-cell function is shown in several studies on ME/CFS patients [25].

Regulation of blood flow in vivo is very complex and involves an array of mediators and the coordinated action of the autonomous nervous system [26]. However, Flow-Mediated Dilation (FMD) executed under standardised conditions adequately reflects the endothelium's ability to produce NO when exposed to increased shear stress from the blood flow [27].

We hypothesise that the symptom maintenance in a subgroup of ME/CFS patients is partly caused by a relative lack of NO bioavailability from endothelial cells. If this is the case, the inadequate regulation of blood flow in response to tissue oxygenation and nutrition requirements will have a fundamental effect on the pattern of symptoms. The next step must be to elucidate what connection there is between the effect of B cell depletion and the effector system with endothelial dysfunction and relative endothelium derived NO unavailability.

The clinical responses after rituximab treatment, starting at the earliest 2 months after the first infusion, suggests the possible involvement of an antibody (long half-life and no

neosynthesis), and the immune response interfering with a signalling pathway which eventually adds up to a disturbed endothelial function, indirectly or directly. If this hypothesis is correct, i.e. if ME/CFS is a variant of an autoimmune disease where an auto-antibody is involved, the existence of a target with an important normal function in regulating endothelial and eNOS activity and possibly also other functions such as uptake of glucose and oxygenation of tissue, might explain the pathogenesis of the disease.

In 1/3 of the patients treated with rituximab there is no clinically significant response. It is possible that other mechanisms independent of the B-lymphocytes can also cause endothelial dysfunction and a similar clinical presentation. We therefore consider the exploration of other principles of treatment an important step towards uncovering adequate treatment options for a larger group of ME patients. Also, by exploring the mechanisms of action in other treatment principles which seem to have effect on the symptoms, we can most likely learn more about the effector systems for symptom maintenance.

Pilot observations of ME/CFS patients with breast cancer

At the Dept. of Oncology at Haukeland University Hospital we have been contacted by two individual patients, both of whom have been diagnosed with breast cancer, with a history of ME/CFS: a relatively mild ME/CFS for 40 years and moderate ME/CFS for 10 years respectively. Both reported considerable effect on their ME/CFS symptoms shortly (days to weeks) after commencing adjuvant chemotherapy for breast cancer according to the FEC treatment protocol, which consists of 5-Fluorouracil, Epirubicine and Cyclophosphamide. Both patients have experienced major and lasting effect on ME/CFS symptoms, and are still in response 6 to12 months after completing 4 cycles of FEC followed by a course of Taxotere cycles.

Pilot treatment with cyclophosphamide in ME/CFS at HUS

Our first observation of considerable beneficial effect of chemotherapy on ME/CFS symptoms was the case of a patient with long lasting ME/CFS who was diagnosed with Hodgkins lymphoma and experienced response following treatment with the MIME regimen. The MIME regimen contains ifosfamide, a chemotherapy drug very similar to cyclophosphamide. Speculations on the B-cell system and the possibility that ME/CFS could be a variety of an autoimmune disease led us to proceed with B-cell depletion using rituximab in 2008.

However, it is quite possible that cyclophosphamide/ifosfamide contributed to the effect on the ME/CFS symptomatology in the above mentioned cases.

Considering our hypothesis (pp 10-12) which involves autoregulation of circulation and the vascular system as a possible effector system for symptom maintenance in ME/CFS, caused by an abnormal immune response in often genetically predisposed individuals, a beneficial effect of cyclophosphamide would not be unexpected. Cyclophosphamide is an important treatment of autoimmune vasculitis, and is also used in rheumatic, inflammatory systemic diseases such as Systemic Lupus (SLE) and Rheumatoid Arthritis, and in Multiple Sclerosis [28-31]. The mechanism of action is not fully known, but the drug is an alkylating agent believed to affect the G1 and S phases of the cell cycle. Cyclophosphamide inhibits both humoral and cell mediated immunity, and is therefore used as a immunosuppressive agent. Based on the two patient cases, our hypothesis on immunodysregulation and the immunomodulating effect of cyclophosphamide, we decided to explore cyclophosphamide as a possible medical treatment of ME/CFS.

In order to gain the necessary experience to design a new open phase II study, we have treated four ME/CFS patients over the last year with up to 6 infusions of cyclophosphamide every four weeks, and have observed the patients closely for any symptom change and/or side effects. Cyclophosphamide was adminstered in doses from 500 mg/m^2 to 700 mg/m^2.
A female patient, 35 years old, with ME/CFS following mononucleosis presented with a history of gradual deterioration over 19 years, and had been housebound and confined to bed or sofa for the last 3 years with a severe ME/CFS. Digital activity registration for 7 days before intervention showed an activity level of a few hundred steps per 24 hours. Approximately 5 weeks after starting infusions of cyclophosphamide, she experienced a gradual improvement in symptoms. The symptom change was unstable during the first months. At 7 months (after completing 6 infusions) her activity level exceeded the 10.000 steps/24 hrs recommended for the general population. During the last few months she has completed walks of over 10 kilometres, at times on a daily basis, and she has been able to swim and cycle. Her lactate related pain is gone, her sleep pattern is nearly normal but the cognitive symptoms are still noticeable, albeit to a lesser extent than at baseline. Occasionally she still suffers ME/CFS symptoms after activity, but she requires less time for restitution. She has now been under observation for 3 months after the final infusion, as yet without relapse.
A male patient, 59 years old, had previously received induction and maintenance treatment with rituximab (KTS-2-2010), but reported increasing symptoms from approx. 1 year after the last infusion. From approx. 4 weeks after the first cyclophosphamide infusion he reported a gradual and significant improvement in symptoms, resulting in a fairly stable function level for the last few months (4 months after completing 6 infusions). He now reports a function level (according to a list of examples) of approx. 65 % of healthy condition. During his best phase after rituximab treatment he reported a function level of 75 %. Before starting rituximab treatment he had relatively severe ME/CFS with a self-assessed symptom level of around 5-10 %.
A 35-year old female with moderate-severe ME/CFS has recently completed six infusions and is experiencing a distinct effect on her symptoms thus far, with less pain, better quality of sleep and higher activity level, but still with a somewhat unstable effect on her fatigue symptoms.
A 49-year old male with very severe ME/CFS, completely bedridden for several years and in need of complete care, only received two infusions, as he was unable to travel to the hospital for further treatments. No significant response was recorded, although the patient felt the treatment did affect his symptoms to some extent.

After some of the treatments the patients experienced adverse reactions such as nausea and general discomfort for a couple of days. No infections, neutropenia, trombocytopenia, alopecia or any other unexpected reactions were recorded.

In our opinion, the preliminary pilot experiences outlined above warrant a phase II trial in order to assess the efficacy, response rates, toxicity, tolerability and feasability of cyclophosphamide infusions in ME/CFS.
Considering the reduced quality of life experienced by most ME/CFS patients, the vast implications of the disease on the patients, their families and next-of-kin, the lack of standard treatment, and the socio-economic consequences of the disease, we believe that it is important to determine whether cyclophosphamide treatment could result in clinically

significant and enduring responses. We need to investigate whether patients who have not responded to rituximab could respond to cyclophosphamide, and whether cyclophosphamide could be a useful option for rituximab responders who may not receive new courses of rituximab (permanently or temporarily). The medication costs are very low, at less than NOK 200 per infusion of cyclophosphamide, and therefore more realistically available than rituximab in areas or settings where economy is a limiting factor.

NEW OPEN PHASE II STUDY: CYCLOPHOSPHAMIDE IN MYALGIC ENCEPHALOPATHY / CHRONIC FATIGUE SYNDROME

Part A, for up to 40 patients with ME/CFS:
An open phase II study with 6 infusions of cyclophosphamide administered at 4 weeks' interval and 18 months follow-up

Part B, for up to 20 patient with severe to very severe ME/CFS:
An exploratory study with up to 6 infusions of cyclophosphamide adminstered at 4 weeks' interval, in collaboration with local health care services

PART A

For up to 40 patients with ME/CFS: An open phase II study with 6 infusions of cyclophosphamide administered at 4 weeks' intervals and follow-up for a total of 18 months.

PROJECT PLAN, ORGANISATION AND COLLABORATION, PART A

Part A of the study will include patients with severe, moderate/severe, moderate and mild/moderate ME/CFS according to the Canadian criteria of 2003.
Part A will be managed by Senior Consultant Øystein Fluge, MD, and Head of Dept. Prof Olav Mella, both at the Department of Oncology and Medical Physics at Haukeland University Hospital (HUS).

For part A, the Clinical Research post at HUS will be responsible for administration of cyclophosphamide infusions and data collection, in collaboration with Olav Mella, Øystein Fluge, dr. Ingrid Gurvin Rekeland and study coordinator Kari Sørland, RN.

Patients living in Eastern Norway, who are included in part A of the study, may receive 4 of the 6 cyclophosphamide infusions at the Norwegian Radium Hospital (DNR) in Oslo, under the supervision of Senior Consultant Alexander Fosså, MD.
Assessment, evaluation, inclusion, registration, examinations (biobank, Sensewear armband registration, endothelial function testing, possibly ergospirometry), first and fourth infusions and follow-up visits at 3, 6, 9 and 12 months will be performed at the study centre at the Dept. of Oncology, Haukeland University Hospital. Cyclophosphamide infusions no. 2, 3, 5 and 6 can be administered at DNR.

The study will be carried out in accordance with the Norwegian regulations: "Regulation relating to clinical trials on medicinal products for human use" (FOR 2009-30-10) and Good Clinical Practice (GCP) guidelines.
The study will be monitored externally by clinical monitor Ingunn H. Anundskås at HUS' Section for patient safety.
We aim to initiate patient inclusion for part A in March 2015, and to include patients over the following 5 months, up to and including August 2015.
Results from part A may be published independently of the plans for trial's part B.

THE MAIN STUDY OBJECTIVE, PART A

The objective of the study’s part A is to assess whether cyclophosphamide infusions every four weeks are associated with clinically significant responses and acceptable toxicity in ME/CFS patients.

ENDPOINTS

Primary endpoint, part A

-Characterization of response will be based on a self-report form (***appendix E1***) which is completed every two weeks during a follow-up period of 18 months after first treatment. Six infusions of cyclophosphamide are given with four weeks’ intervals, 600 mg/m^2 at first infusion and 700 mg/m^2 at second and later infusions if tolerance is acceptable.

The variable **Fatigue score** is registered every two weeks as the mean score (scale 0-6) for the four fatigue-related symptoms: ”Post-exertional malaise”, ”Fatigue”, ”Need for rest” and ”Daily function”.
The course of Fatigue score throughout follow-up is described. The scale for registration is 0-6, where 3 represents no change from baseline, 4-5-6 means slight, moderate and major improvement and 2-1-0 represents slight, moderate and major worsening respectively. The 0-6 scale is relative, and always requires comparison with status at baseline, before first intervention.

The primary endpoint will be change of self-reported Fatigue score from baseline to mean score for each of the time intervals 0-3, 3-6, 6-9 and 9-12 months during follow-up, as an expression of the effect on ME/CFS symptoms.
Equivalent analyses will be performed for the course throughout follow-up for self-reported changes in Cognitive score, Pain score and Sleep ***(appendix E1)***.
Data may be compared with historical controls from the placebo group in the completed and published, randomised phase II trial KTS-1-2008.

The Overall Response records the effect on the ME/CFS symptoms during 12 months after intervention start date. The Overall Response is not predefined to a specific time interval during the 18 months of follow-up, but the response must be recorded as moderate or major on the patient self-report form. Overall Response is defined as mean Fatigue score ≥ 4.5 for a minimum of 6 consecutive weeks for moderate response, and including a mean Fatigue score ≥ 5.0 for a minimum of 6 consecutive weeks for major response. The duration and sum of the various response periods during the 18 months will be recorded.

Response data will be reported separately for the 25 “naive” ME/CFS patients (who have not received previous rituximab intervention). Response data according to primary endpoints will also be reported separately for patients who have previously received rituximab without clinical effect, and for patients who have previously experienced clinical effect and relapse after rituximab. Response according to primary endpoints will also be reported for all included patients in total.

Secondary endpoints, part A

- SF-36 scores (”Physical health summary score”,”Mental health summary score” and scores for eight SF-36 subdimensions) are analysed at baseline and at 3, 6, 9, 12, 15 and 18 months ***(appendix F1)***.
Changes in the SF-36 ”Physical health summary score” (norm based), the SF-36-subdimension ”Physical Function” (raw score) and changes in mean scores for the five SF-36 subdimensions ”Physical Function”, ”Bodily Pain”, ”Vitality”, ”Social Function” and ”General health” (raw scores), from baseline to 3, 6, 9, 12, 15 and 18 months, will be recorded.

- Changes in physical activity measured by a Sensewear armband for seven consecutive days, before intervention and again between 7 and 9 months after intervention start date, after 11 to 12 months and after 17 to 18 months. Changes will be recorded for mean number of steps per 24 hours, max. number of steps per 24 hours, mean duration of moderate activity ≥ 3.5 METs per 24 hours, max. duration of moderate activity ≥ 3.5 METs per 24 hours.

- Patients who are deemed physically capable of completing an ergospirometry stress test, will do so for two consecutive days before medical intervention and again after 7 to 9 months, and possibly repeated after 11 to 12 months. Tests are performed using a bicycle ergometer with a programmed ramp protocol with increases in wattage of either 10 Watt/min, 15 Watt/min, 20 Watt/min, 25 Watt/min or 30 Watt/min, depending on clinical assessment, gender and symptom severity.
Oxygen uptake and work load (Watt) on day two, at maximum work load and anaerobic threshold, will be compared to the equivalent values at 7 to 9 months after start intervention and possibly also after 11 to 12 months. Changes from baseline (before intervention) to the repeated stress test at 7 to 9 months (and 11 to 12 months) will be recorded

--”Total function level” (scale 0-100, compared to healthy state) is recorded in the patient’s self-report form ***(appendix E1)*** every two weeks.
Changes in ”Total function level” from baseline to the mean score for each of the time intervals 0-3, 3-6, 6-9, 9-12, 12-15 and 15-18 months are recorded.

-The Fatigue Severity Scale (FSS) will be completed at baseline, and at 3, 6, 9, 12, 15 and 18 months. Changes in FSS score from baseline and throughout follow-up will be recorded (***appendix F2***).

-The longest duration of lasting clinical response defined as lasting self-reported Fatigue score ≥ 4.5 (at least 6 consecutive weeks) during the 18 month follow-up period is recorded.

-The number of patients who have recorded response according to the response criteria and who show no sign of relapse (lasting Fatigue score ≥ 4.5 at 18 months follow-up), will be registered.

-Toxicity throughout 18 month follow-up.

In part A at least 25 included patients will be “new” (treatment naïve) ME/CFS patients who have not previously taken part in clinical trials with rituximab. We also plan the inclusion of patients classified as non-responders after rituximab intervention as well as patients with a

clear clinical response but partial or complete relapse after previous rituximab intervention during the KTS-1-2008 and/or KTS-2-2010 trials. In total up to 40 patients will be included in part A. For patients who have taken part in previous clinical trials, the response data after cyclophosphamide intervention may be compared to the equivalent historical data from patient self-report during these trials.

Response data for the secondary endpoints will be reported separately for the 25 “naive” ME/CFS patients (who have not received previous rituximab intervention). Response data according to secondary endpoints will also be reported separately for patients who have previously received rituximab without clinical response, and for patients who have previously experienced clinical response and relapse after rituximab. Response according to secondary endpoints will also be reported for all included patients in total.

DESIGN, PART A

Open phase II study.

PATIENT SAMPLE, PART A

Up to 40 patients will be included in part A. Patients must have an established ME/CFS diagnosis according to ”Canadian criteria” [8] ***(appendix A).***
The patients must be between 18 and 66 years old, with a minimum disease duration of two years. The patients may suffer from Mild/Moderate, Moderate, Moderate/Severe or Severe ME/CFS.
Patients with mild ME/CFS or very severe ME/CFS (bedridden and in need of care, WHO class IV) will not be included in part A.

A joint assessment by both investigator and patient must conclude that ME/CFS severity and loss of function in each individual patient justify the medical intervention.

In part A, at least 25 of the included patients must be treatment naïve, i.e. patients who have not previously received rituximab intervention for ME/CFS.
We also plan the inclusion of patients classified as non-responders after rituximab intervention (one third of patients in the KTS-1-2008 and/or KTS-2-2010 trials) as well as patients with a clear clinical response but partial or complete relapse after previous rituximab intervention.
Response rates for part A will be reported for all patients in total and for the three groups separately (treatment naïve, rituximab non-responders, relapsed rituximab responders).
In the group of 25 treatment naïve patients a response rate of 36% will yield a 95% CI 20%-55%, for a response rate 52% the 95% CI will be 34%-70%, and a response rate of 72% will yield a 95% CI of 52%-86%.

INCLUSION CRITERIA, PART A

- Patients with ME/CFS according to Canadian criteria of 2003 [8].
- Disease duration of minimum two years.
- Severity: Mild/Moderate, Moderate, Moderate/Severe or Severe ME/CFS.
- Age 18 - 65 years.
- Signed informed consent.

EXCLUSION CRITERIA, PART A

- Patients with fatigue, who do not comply with the diagnostic (”Canadian”) criteria for ME/CFS or disease duration < 24 months
- Patients with mild degree of ME/CFS.
- Patients with very severe ME/CFS (bedridden and in need of care).
- Patients where the workup uncovers other pathology as a possible cause of symptoms.
- Pregnancy or breast feeding. Positive pregnancy test.
- Previous cancer (except basal cell carcinoma of the skin or cervix dysplasia).
- Previous long-term systemic treatment with immunosuppressive agents (Imurel, Sandimmun, Cellcept), except steroid treatments for e.g. obstructive lung disease or other autoimmune diseases like ulcerative colitis.
- Serious endogenous (primary) depression.
- Lack of ability to complete the study including follow-up.
- Reduced kidney function (creatinine > 1.5 x reference area).
- Reduced liver function (bilirubin > 1.5 x reference area, or transaminase > 1.5 x reference area).
- Severely reduced bonemarrow function.
- Ongoing cystitis or obstructive uropathy
- Known HIV positivity, previous hepatitis B or hepatitis C, or reason to suspect other ongoing and clinically relevant infection.

CONSULTATION, PART A

The patients in the study’s part A will attend the Department of Oncology at Haukeland University Hospital consultation and assessment, and distribution of a written patient information letter/declaration of consent. No study specific analyses or tests should be performed prior to written informed consent from the patient.
In accordance with the protocol, candidates will be invited for clinical assessment, laboratory and other tests prior to intervention, such as blood samples for basic workup and for the biobank, and Sensewear activity registration for 7 consecutive days.
Male patients will be offered the opportunity for cryopreservation of sperm before start of intervention.
Patients will then attend the relevant examinations, such as endothelial function tests (FMD and microcirculation) ***(appendix H1)*** and ergospirometry for two consecutive days – for patients who are deemed physically capable of undergoing such testing ***(appendix H2)***.

INTERVENTION, PART A

Cyclophosphamide infusions are administered at the Clinical Trial Unit at Haukeland University Hospital. Patients who are resident in Eastern Norway, may receive some of the infusions at the Norwegian Radium Hospital in Oslo.

Intravenous infusions of cyclophosphamide are administered every four weeks. A margin of +/- four days is allowed, i.e. an infusion should be administered 25 to 33 days after the previous infusion.
The initial infusion will contain a dose of cyclophosphamide 600 mg/m^2, and if the treatment is adequately tolerated with no significant impact on hematological parameters (i.e. neutrophils > 1.5 and thrombocytes > 100 before next treatment) the dose will be increased to 700 mg/m^2 for the following infusions.

After the first and second infusion a blood sample will be collected during expected nadir phase, i.e. 10 to14 days after infusion, registering hemoglobin, leukocytes, neutrophils and thrombocytes. If there is no sign of neutropenia (< 1.0) or thrombocytopenia (< 75) during nadir after the first two treatments, no further blood samples between treatments will be required. A new blood sample must always be collected zero to three days before next treatment (patients may subject to blood sample control on the same morning as the infusion if feasible).
During part A, a total of six cyclophosphamide infusions are given with four week intervals.

In order to prevent nausea, 8 mg of ondansetron are administered morning and evening on the day of treatment and the following day. If the patient suffers nausea after the first two days, 8 mg ondansetron may be used once or twice a day for another 2 days, in combination with 10 mg metoclopramide up to 3 times per 24h if required.

Intravenous infusions will be prepared according to standardised pharmacy procedures, with study specific labelling and drug accountability records.

All cyclophosphamide infusions are administered as follows:
Premedication (8 mg ondansetron) is administered orally 60 min. before or IV 30 min. before start of cyclophosphamide. An IV cannula is inserted into a peripheral vein, and 500 ml NaCl 0.9% is administered during 20 min., followed by cyclophosphamide diluted in 250 ml NaCl 0.9% during 15 min, and eventually 500 ml NaCl 0.9% during 20 min. The total infusion time is approximately one hour, but may be adjusted as required. The patients may choose to wear a cooling cap from 15 min. before to 15 min. after completed cyclophosphamide infusion in order to prevent hair loss. A doctor must be available in the building during infusions.

DATA COLLECTION AND DATA MANAGEMENT, PART A

Data manager is study coordinator Kari Sørland, in collaboration with the Clinical Trials Unit, HUS. Each included patient is registered by study coordinator Kari Sørland or the Clinical Trials Unit, and is allocated a unique study ID number (e.g. 7A-04, denoting part A, patient number 4).

The patients will receive individual study folders. The study folder must be brought to each visit at the trial site. The folder contains (separated by partitions) a front page with contact information and a ”checklist/calendar”, a copy of the written patient information/consent form, a form for self-reporting of symptoms at baseline (scale 1-10), a form for self-reporting of symptom change (scale 0-6) and total function level (scale 0-100) every two weeks during follow-up, SF-36 quality of life questionnaire to be completed after 0, 3, 6, 9, 12, 15 and 18

months and Fatigue Severity Scale (FSS) questionnaire to be completed after 0, 3, 6, 9, 12, 15 and 18 months.

At every clinical assessment visit, the relevant pages of the self-report form (completed every two weeks since last visit) are photocopied. The original remains in the patient folder. Completed SF-36 and Fatigue Severity Scale (FSS) forms are collected from the patient folder at relevant time points (no copy in patient folder). The collected original forms and copies of self-report form are stored in the patients' individual case file at the trial site. All original forms are collected at end of study, and are considered source documents. Documents will contain no personal data, but will be linked to patient data through the unique study ID number.

Data from the forms will be entered into the program Viedoc®, a designated eCRF system for clinical studies which comply with all requirements from the Data Protection Office, the Norwegian Medicines Agency (NOMA), and international agencies such as the US Food and Drug Administration (FDA).

The Clinical Trials Unit at HUS will be responsible for data input.
Study nurses at the Clinical Trials Unit are well versed in Good Clinical Practice (GCP) and will handle patient flow and data management during the treatment phase. The study management at Haukeland University Hospital (Olav Mella, Øystein Fluge, Ingrid Gurvin Rekeland, Kari Sørland and the Clinical Trials Unit) will have access to registered data from all included patients.
Paper copies are stored at the study centre. After study completion the patients' study folders including the original self-report form will be archived according to regulations. Original documents will be stored at the trial sites for 15 years after final report is issued. All computer files, including the patient enrollment lists, will be stored on a study specific area of the HUS research server for 15 years. Data from clinical visits will be entered in the hospital's electronic medical journal system.
Study data will be handled according to a study specific data management plan.
SPSS and Graphpad Prism may be used for statistical analysis.
Patient groups will be characterized for demographical and clinical data. We will register changes over time for the individual patient and for the group in total. Patients will be assessed for statistically and clinically significant changes.

Modified DePaul and HADS questionnaires

Before inclusion in part A of the study, all patients will complete a modified DePaul questionnaire ***(appendix B)***.
Patients also complete the Hospital Anxiety and Depression Scale (HADS) form for evalueation of symptoms related to anxiety and depression (***appendix F3***). The HADS questionnaire consists of 7 questions related to anxiety and 7 questions related to depression, with four alternative answers (scale 0-3) for each question [32]. HADS is thoroughly validated and frequently used in studies in order to clarify any existing component of anxiety or depression. The patients will only complete the HADS questionnaire at baseline as part of the workup before intervention.
The modified DePaul and HADS forms must be completed by all patients included in part A, but may be omitted for patients with very severe illness in part B of the study.

Self-reported symptom score

As there are no definite diagnostic laboratory tests or other specific markers for the disease, patient symptoms are key variables for adequate registration of clinical effect. The effect variables are related to the four main symptom categories in ME/CFS: ”Fatigue”, ”Pain”, ”Cognitive symptoms”, and ”Other symptoms ” (including sleep disturbances, sensory hypersensitivity and symptoms from the autonomous nervous system).

Self-reported symptom score before intervention

Each patient will complete a registration form (after inclusion, before intervention) with scores (scale 1-10) for the patient’s present symptoms, and a score for «Total function level» which is stated as a percentage of a completely healthy state (i.e. 100 %), guided by a set of examples in the patient’s study folder ***(appendix D1)***.
Only symptoms which are relevant for the individual patient (i.e. it transpires from the registration form that these symptoms are actually affecting this particular patient) will be analysed for changes during follow-up.

Self-reported symptom change during follow-up

Each patient in part A will fill in the self-report form for symptom change every two weeks, until 18 months follow-up is complete. Symptom change is compared to status before intervention (baseline) throughout the follow-up period.

Changes in symptom severity (scale 0-6, where 3: unchanged, 4: slight improvement, 5: moderate improvement, 6: major improvement, 2: slight worsening, 1: moderate worsening, 0: major worsening), are always stated as compared to status before intervention, and will be completed by the patients every 2 weeks throughout the follow-up period ***(appendix E1)***.

A ”symptom score” for each of the four main symptom categories ”Fatigue”, ”Cognitive symptoms”, ”Pain” and ”Other symptoms” expresses the mean score for the symptoms under each category recorded every two weeks on the self-report form.

The Fatigue score is recorded every two weeks as the mean score for the following four parameters: Fatigue, Post-exertional malaise, Need for rest and Daily function.

The Cognitive score is recorded every two weeks as the mean score for the following three parameters: Concentration difficulties, Memory problems, and Ability to think clearly.

The Pain score is recorded every two weeks as the mean score for the following symptoms: Muscle pain, Joint pain, Headache and Skin pain, provided that the patient actually suffers from the specific pain symptom (preregistration at baseline ≥ 5, scale 1-10).

The Sleep score is recorded every two weeks for the variable Sleep problems.
For the category ”Other symptoms” score, changes are recorded for the two symptoms perceived as characteristic for the individual patient’s ME/CFS, out of the five symptoms in this category with the highest score on the preregistration form at baseline.

An average for each symptom score (Fatigue score, Cognitive score, Pain score, ”Other symptoms” score) is calculated for the time intervals 0-3, 3-6, 6-9, 9-12, 12-15 and 15-18 months during the follow-up period [10].

Changes in mean Fatigue score from baseline to the specified intervals form the basis of the primary endpoint.

”Total function level"

Changes in self-reported symptom scores compared to baseline will be relative, as major improvement (i.e. value 6, scale 0-6) will be perceived differently by a patient who is somewhat active and on their feet prior to intervention, and a patient who is seriously ill and mainly bedridden prior to intervention.
Therefore, the patients will estimate their «total function level» every two weeks, as a percentage of a totally healthy state before symptom debut (which corresponds to 100 %), according to the sheet of examples in the patient folder ***(appendix D1).***

Mean "Total function level" over the last 3 months is recorded at 3, 6, 9, 12, 15 and 18 months. The change in Fatigue score from baseline to the mean score for each time point is registered. An equivalent self-report form has been used in the KTS-1-2008 [10] and KTS-2-2010 trials, as well as in the ongoing randomised phase III RituxME trial.

SF-36 questionnaire on health, and analysis

-The Short Form 36 (SF-36) questionnaire on health will be completed by patients in part A before intervention and at 3, 6, 9, 12, 15 and 18 months ***(appendix F1)***.
At these visits, a completed SF-36 questionnaire is handed in (no copy in patient folder).
SF-36 v1.2 is a generic (diagnosis-independent) form which is widely evaluated [33,34]. We use a Norwegian validated translation [35].
In the SF-36 questionnaire for follow-up, question 2: “Compared to one year ago, how would you rate your health in general now?” has been replaced with: “Compared to before the start of the study, how would you rate your health in general now?”

The SF-36 is analysed using a standardized SPSS syntax file, where the results for ”Physical health summary score” and ”Mental health summary score” are interpreted using norm-based scoring (population mean = 50), and the results for the eight SF-36 subdimensions can be expressed either as ”raw scores” (scale 0-100) or as norm-based scores (US 1998).

The SF-36 ”Physical health summary score” (norm-based) and the SF-36 subdimension ”Physical Function” expressed as a raw score (scale 0-100), plus the mean SF-36 raw scores for the five subdimensions (”Physical Function”, ”Bodily Pain”, ”Vitality”, ”Social Function” and ”General Health”, scale 0-100), at 0, 3, 6, 9, 12, 15 and 18 months, will be used for statistical analysis. The analysis will process the change in the equivalent SF-36 scores (“Physical health summary score”, “Physical Function raw score”, and the mean of the five subdimensions (PF, BP, GH, V, SF) from baseline to each of the time points 3, 6, 9,12, 15 and 18 months.

Fatigue Severity Scale

The Fatigue Severity Scale (FSS) form consists of 9 questions related to fatigue, where each item is scored from 1 (completely disagree) to 7 (completely agree), and the patient’s FSS score equals the mean score for the 9 items. FSS has been used in a variety of studies on ME/CFS patients (***appendix F2***).

The patients in part A will complete the FSS at baseline and 3, 6, 9, 12, 15 and 18 months after start of intervention.

At relevant visits the completed form is handed in and stored in the patient case file. Change in FSS scores from baseline to registration at 3, 6, 9, 12, 15 and 18 months is recorded.

Doctor’s registration at baseline and follow-up

At baseline assessment, the doctor will assess the ME/CFS severity for each patient: Mild, Mild/Moderate, Moderate, Moderate/Severe, or Severe (***appendix G***). Patients with Mild or Patients with Mild or Very severe ME/CFS are not eligible for part A.
The investigator must grade the different symptoms at baseline before intervention (scale 1-10) and record these on the form.
Registration of symptom change at follow-up (scale 0-6) and toxicity assessment will be performed at visits at 3, 6, 9, 12, 15 and 18 months and recorded on the form ***(appendix G)***.
After the formal study follow-up period (18 months) we will attempt to maintain contact with the patients for at least 5 years in order to capture any long term effect or toxicity.
Patient drug records will be registered. Any dietary supplements must be recorded, and during the study period the patients may not start taking any new supplements without consulting a study doctor.
Any side effects including infections are recorded. Each visit must be documented in the patient’s electronic journal.

Sensewear armbands for recording activity level at home

The patients’ level of physical activity measured with a Sensewear armband for 7 consecutive days will be recorded after inclusion in part A of the study, and before start of intervention with cyclophosphamide. Activity registration with a Sensewear armband must take place before ergospirometry where this is relevant. The registration is repeated for 7 consecutive days during the time interval 7-9 months, 11-12 months and 17-18 months after start of intervention.
Sensewear armbands are validated and used to assess physical activity in patients with rheumatoid arthritis, and is considered suitable for monitoring changes in patient physical activity after interventions [36,37].
The Sensewear armband will be distributed with a letter of information to all study participants from the trial site at Haukeland University Hospital with a return envelope for return to HUS after 7 days registration. The information on all armbands will be downloaded and analysed at the Oncology Dept., HUS.

Based on the analyses of Sensewear data for ME/CFS patients in the completed clinical study KTS-2-2010, the change in mean number of steps per 24 hours, the change in mean number of steps per 24 hours, the change in mean duration (≥ 3,5 METs), and the maximum duration of moderate activity level (≥ 3,5 METs) per 24 hrs are registered.

Other variables from the Sensewear armbands include:
Mean per 24 hours for: total energy expenditure, mean METs, time for physical activity (expressed as sedentary/light activity < 3,5 METs, moderate activity 3,5-6,0 METs, vigorous activity >6,0 METs), total duration of physical activity >3,5 METs, time lying down, sleep

duration, duration on-body per 24h (armband must in principle be worn at all times except during bath/shower).

Changes from baseline to the time intervals 7-9 months, 11-12 months and 17-18 months are analysed.

EXAMINATIONS AND REGISTRATION BEFORE INTERVENTION, PART A

Clinical assessment

The doctor assessing the patient will check the inclusion and exclusion criteria and decide whether there is any need for supplementary testing. The assessment will involve exclusion of other medical conditions which may cause considerable fatigue such as: hypothyreosis, adrenal insufficiency, malignity, chronic infections, lung disease, angina pectoris, heart failure, kidney failure, liver disease, other neurological diseases (multiple sclerosis, brain tumours or cerebrovascular disease), endogenous depression or other psychiatric conditions associated with fatigue.

The doctor will assess the ME/CFS symptomatology and record the severity of the symptoms (Mild/Moderate, Moderate, Moderate/Severe, and Severe) on the relevant form ***(appendix G1)***.

-The patient will complete several questionnaires for assessment of symptoms; the modified DePaul questionnaire ***(appendix B)***, the SF-36 quality of life questionnaire (***appendix F1***), the Fatigue Severity Scale (FSS) questionnaire (***appendix F2***) and the Hospital Anxiety and Depression Scale (HADS) questionnaire (***appendix F3***).
-The modified DePaul, SF-36, FSS and HADS questionnaires shall be handed in after completion and will be stored in the patient's case file at the trial site.

-The patients will also complete the self-report form for symptoms at baseline (scale 1-10) and the "Total function level" (0-100 %, in accordance with the instructions in the study folder (***appendix D1***)). A copy of the self-report form is stored in the case file, while the original stays in the patient's study folder.

- Candidates may not be included while undergoing Gammanorm® or other immunoglobuline treatment. Subcutaneous immunoglobuline injections must be discontinued at least three months before inclusion in the study.
- Treatment with low dose Naltrexone (LDN, up to 4.5 mg/day) which was initiated less than three months before clinical assessment should be discontinued. If the treatment has been ongoing for more than three months, the patient may continue the LDN treatment throughout the study. Patients who have not previously used LDN may not start this treatment during the study period.
- Patients should not start new dietary supplements during the study period; this particularly applies to Arginine or Citrulline supplements. If the patient has been using the supplements for at least three months before inclusion, they may continue the supplements throughout the study period.

- Treatment with GcMAF or Isoprinosine must be discontinued at least three months before inclusion in the study.
- Treatment with vitamin B12 which was initiated less than three months before clinical assessment should be discontinued. If the treatment has been ongoing for more than three months, the patient may continue the B12 treatment throughout the study.
- Common vitamin supplements, fish oil, omega-3 and similar over-the-counter supplements are acceptable.

LABORATORY TESTS AT BASELINE, PART A

<u>Any blood tests featured under Immunology, Endocrinology and/or Microbiology (see below) which have already been performed over the previous 6 months, need not be repeated at baseline.</u>

Routine laboratory tests

- Hb, ESR, WPC differential, Platelet count, MCV.
- Ferritin, Fe, TIBC, Vitamin B12, Folate, Na, K, Ca, Mg, Phosphate, Glucose.
- Creatinine, Urea, Urate, Total Cholesterol, HDL Cholesterol, 25-hydroxy-vitamin D.
- ALT, ALP, GGT, Bilirubin.
- CRP, Albumin, Total protein, INR.
- HCG for women of childbearing age.
- Urine dipstick test.

Immunology

- Serum Protein Electrophoresis, Quantitative Immunoglobulins with IgG, IgG subclasses, IgM, IgA.
- Immunophenotyping of mononuclear cells in peripheral blood (lymphocyte quantification). This sample must be sent to the Dept. of Transfusion medicine and Immunology at HUS, att.: Head of Dept. prof. Einar K. Kristoffersen, labelled "Cyclophosphamide study") ***(appendix I)***.
- tTGA (Celiac Disease Test), Antinuclear Antibody Test, anti-CCP, Thyroid Antibodies (Anti-TPO), Cardiolipin antibodies.

Endocrinology

-FT4, TSH, Prolactin, Cortisol/ACTH.

Microbiology

-Serology for EBV, CMV, Parvovirus B19, Borrelia, HIV, Hepatitis serology (HBV, HCV).

Biobank blood samples

(see Appendix I)

SENSEWEAR ARMBAND FOR ACTIVITY REGISTRATION AT BASELINE, PART A

The Sensewear armband will be mailed to all study participants from the trial site at HUS, along with a letter of instructions and a stamped return envelope to be used after the 7 day registration period.
We aim to complete activity registration for all patients at baseline, for both part A and B of the study.

ENDOTHELIAL FUNCTION (FMD) AND MICROVASCULAR FUNCTION AT BASELINE, PART A

Tests for endothelial function (Flow-mediated Dilation, FMD) and microvascular function (laser Doppler/Periflux 5000) will be performed before intervention in part A of the study ***(vedlegg H1)***.

ERGOSPIROMETRY AT BASELINE, PART A

Ergospirometry testing will be performed for patients with a mild/moderate or moderate degree of ME/CFS, or if possible selected patients with moderate/severe or severe degree of ME/CFS, where the patient and the doctor agree that the patient is capable of performing the test without undue risk. The test must be performed after clinical assessment, all blood samples, Sensewear activity registration and endothelial function testing have been completed.
A minimum of three weeks must pass from the completed ergospirometry to the start of cyclophosphamide intervention, as the patient's condition may deteriorate temporarily after a physical exercise test – in particular in patients with moderate/severe to severe degree of ME/CFS. Ergospirometry is performed with an identical setup on two consecutive days, before intervention and again at 7-9 months follow-up, and possibly repeated after 11-12 months ***(see appendix H2).***

CYCLOPHOSPHAMIDE INFUSIONS EVERY FOUR WEEKS, PART A

The patients will receive a total of 6 cyclophosphamide infusions at four week intervals. Each infusion will be preceded by a basic patient consultation, including a simple clinical assessment if required due to new symptoms/side effects. The patient consultations and assessments are carried out by a study nurse at infusions no. 2, 3, 5 and 6.
The consultations and assessments at 3, 6, 9, 12, 15 and 18 months are carried out by investigator. CRFs will be collected at these visits as detailed above.

Routine blood tests are run before each cyclophosphamide infusion:
Hb, WPC differential, Platelet count, Na, K, Creatinine, Bilirubin, ALT, ALP, GGT. Before infusion the tests must show neutrophile granulocytes > 1.5 and platelets > 100. A urine dipstick test is performed.
After the first and second cyclophosphamide infusions a nadir blood sample (Hb, WPC differential, Platelet count) is taken between day 10 and 14. If there is no sign of neutropenia (< 1.0) or thrombocytopenia (< 75) during nadir after the first two treatments, no further blood samples between treatments will be required.

EXAMINATIONS AND REGISTRATION AT 3, 6, 9, 12, 15 AND 18 MONTHS, PART A

Clinical assessment with registration and entry in medical records

At each visit the doctor will perform a clinical assessment and dictate an entry in the patient's electronic records.
The patient's completed self-report form (symptom change (scale 0-6) and total function level (scale 0-100), recorded every two weeks – ***appendix E1***) for the relevant time period is

copied and stored in the patient's case file. The original form stays in the patient's study folder.
The doctor will verify the patient's record of any symptom change, (scale 0-6) on a separate form ***(appendix G1)***.

The SF-36 and the FSS questionnaires for the relevant visit are collected and stored in the patient's case file (no copy in patient folder).

Laboratory tests

-Hb, ESR, WPC differential, Platelet count, Na, K, Ca, Phosphate, Glucose, Creatinine, Urea, Urate, ALT, ALP, GGT, LD, Bilirubin, CRP, Albumin, Total protein. Urine dipstick test.

Blood samples for biobank

Blood samples for the biobank are collected at 6, 12 and 18 months ***(appendix I).***

SENSEWEAR ARMBAND FOR ACTIVITY REGISTRATION AT 7-9, 11-12 AND 17-18 MONTHS

The Sensewear armband will be handed out or mailed to all part A participants from the trial site at HUS.
We aim to complete 7 day activity registration at each timepoint, for both part A and B of the study.
The armband will be accompanied by a letter of instructions and a stamped return envelope.
Download and analysis of all Sensewear data takes place at the Oncology Dept. at HUS.

ENDOTHELIAL/MICROVASCULAR FUNCTION TESTS AT 7-9 AND/OR 11-12 MONTHS

Tests for endothelial function (Flow-mediated Dilation, FMD) and microvascular function (laser Doppler/Periflux 5000) will be performed 7-9 months after intervention in part A of the study, alternatively also at 11-12 months ***(vedlegg H1)***.

ERGOSPIROMETRY AT 7-9, ALTERNATIVELY AT 11-12 MONTHS

Ergospirometry testing at 7-9 months follow-up, alternatively at 11-12 months, will be performed for patients with a mild/moderate or moderate degree of ME/CFS, or possibly selected patients with moderate/severe of ME/CFS, who completed such testing at baseline ***(appendix H2)***.

VISIT AFTER 12 MONTHS, AND FINAL VISIT AFTER 18 MONTHS.

At the 12 and 18 month visits the pasient will be requested to state in the self-report form ***(appendix E1)*** an overall assessment of any change of ME/CFS symptoms throughout 12 months and 18 months follow-up. This assessment should not simply express the patient's status at the 12 or 18 month visit, but reflect the overall development over the study period.

At 12 and 18 month visits patient must choose one of the following categories:
1. Worsening of ME/CFS symptoms during the 12 (18) month study period
2. No significant change in ME/CFS symptoms during the 12 (18) month study period, beyond habitual symptom variation

3. Moderate improvement of ME/CFS symptoms during the 12 (18) month study period
4. Major improvement of ME/CFS symptoms during the 12 (18) month study period

Whether a patient should record any improvement as moderate or major, will depend on the degree and duration of the improvement, but does not depend on the patient's continued response at 12 (18) months.

FOR BOTH PARTS A AND B OF THE STUDY:

MONITORING

The study will be monitored continually by external monitor Ingunn H. Anundskås, Innovest AS (Bergen). Investigators will allow direct access to source data including entries in the electronic patient journal, during monitoring, audit or inspection from the Norwegian Medicines Agency (NoMA).

BIOBANK FOR BIOLOGICAL STUDIES AND IMMUNOPHENOTYPING

Blood samples for all patients who have been included in clinical studies of ME/CFS at the Dept. of Oncology, HUS, have been collected at baseline and throughout follow-up and stored in a biobank. We will systematically extend the existing approved biobank at HUS with biological material from patients included in this study, before intervention and throughout 18 months of follow-up.

Existing biobank: *Medical intervention for chronic fatigue syndrome.* Responsible: Olav Mella. Approved by REK prior to May 5th, 2009. Project number 5.2008.67. Case/file number 2998000657-9/MRO/400. Form number in previous database notification 2219. EudraCT number 2007-007973-22. Approved by the Norwegian Directorate of Health. Physical location: Department of Oncology and Medical Physics, Haukeland University Hospital. For each project and clinical study regarding ME/CFS at the Dept. of Oncology at Haukeland University Hospital during 2009-2014, applications to the Regional Ethical Committee have included applications for extending the existing biobank.

The biobank will be a starting point for further research into the pathogenesis of ME/CFS. The mechanisms behind the disease must be charted, and a specific and sensitive biomarker is greatly needed.
In addition to laboratory tests as specified above, before treatment and at follow-up after 3, 6, 9, 12, 15 and 18 months, blood samples for biobank and research will be collected at baseline and after 6, 12 and 18 (informed consent obtained before inclusion).
For patients not resident in Bergen, complete sets of pre-labelled blood collection tubes may be sent from HUS to the doctor/nurse responsible for follow-up. The samples must be forwarded to the central biobank at the Dept. of Oncology, Haukeland University Hospital (***appendix I***).

At baseline and after visits at 6, 12 and 18 months an EDTA tube (3 ml) of whole blood shall also be sent to the Dept. of Transfusion Medicine and Immunology at HUS, att.: Head of Dept. Prof. Einar K. Kristoffersen, for immunophenotyping of lymphocyte populations in peripheral blood. This sample must be stored and shipped at room temperature, and must arrive at HUS within 3 days. The tube must be labelled ”Cyclophosphamide study”, the patient’s study ID number and number of months after intervention ***(appendix I)***.

Additional samples are optional, and may include a punch biopsy from skin/underlying tissue on the thigh for formalin fixation, a true-cut needle biopsy from the lateral thigh muscle for protein purification, RNA purification and paraffin-embedding and/or cerebrospinal fluid for freezing, before intervention and possibly repeated at 7-9 and/or 11-12 months.

PATIENT WITHDRAWAL DURING STUDY

The patients will be informed verbally and in the written patient information that they may withdraw from the study at any time, without having to state the reason for their decision. The medical reasons for withdrawing a patient from the study may be serious events such as severe reactions during or short time after the infusion. Patients who withdraw from the study due to intercurrent or other diseases or any other reason will be followed by their general practitioner according to usual ME/CFS guidelines. If possible, we will attempt to obtain toxicity data from patients who have withdrawn during the study.

If a patient is included in the study, but withdraws before intervention or for other reasons does not receive the medical intervention, this patient may be replaced by another patient.

We will aim to replace patients who drop out after inclusion, so that at least 25 treatment naïve patients (who have not received rituximab previously) and a total of 40 patients receive the study medication in part A. Part B requires a more flexible approach, as some patients with very severe disease may not succeed in completing the planned intervention. We will follow these patients as closely as possible in collaboration with the local primary health care services in order to register data on response, toxicity and feasibility. We will aim to replace any dropouts in part B as well, but recognize that the inclusion process will be challenging. The goal is to include up to 20 patients.

ADVERSE EVENTS, SAFETY BOARD, SIDE EFFECTS

The study management is responsible for follow-up on any reported side effects during the study period; in part B of the study in collaboration with the local investigator. The local investigator will report any serious adverse events to the central study management at HUS, who are responsible for further reporting to the Regional Ethical Committee and the Norwegian Medicines Agency (NOMA).

AEs (Adverse Events) will be reported in the eCRF for part A, and a paper form for part B.

SAEs (Serious Adverse Events) are defined as Adverse Events which are deadly or life-threatening, result in hospitalization (initial or prolonged), lasting or significant disability or incapacity or a congenital anomaly or birth defect, or is considered serious for other reasons. SAEs must be reported on a separate form ***(appendix K)*** and sent to sponsor (Haukeland University Hospital, att.: Head of Dept. Olav Mella) within 24 hours after the trial site being notified of the event.

A SUSAR (Suspected Unexpected Serious Adverse Reaction), is defined an SAE which is suspected related to the trial medication, and is also unexpected.
SUSAR must be reported to NOMA according to the following guidelines: all deadly or life-threatening SUSARs must be reported to NOMA immediately and within 7 days of sponsor being notified of the event. Other SUSARs must be reported to NOMA within 15 days.

Only SUSARs will be reported as an individual report. The responsibility for reporting of SUSARs to NOMA lies with the central study management at the Oncology department at HUS.
The remaining adverse events will be reported in a combined final report. The central study management at HUS is responsible for annual reporting of serious adverse events to NOMA.

Changes in ME/CFS symptoms during the follow-up period and temporary worsening of symptoms during the weeks immediately following the rituximab infusions will not be recorded as Adverse Events. Peroral antibiotic treatments over a shorter period of time for otherwise uncomplicated infections (URI, lower UTI) are recorded and reported in the combined final report.

Safety board and safety profile

The safety aspect of the study will be supervised by a Safety Board. Members are Prof. Olav Dahl, Dept. of Oncology, Haukeland University Hospital (Chairman) and Prof. Ola Didrik Saugstad, Dept. of Paediatrics, Rikshospitalet OUS.
The Safety Board will monitor Adverse Events (AE) and Serious Adverse Events (SAE) in the study.
Members of the Safety Board will not take part in the clinical assessment of patients.

Part A is an open phase II study. Part B is also an open phase II study of a more explorative character, as the feasibility of this intervention for patients with very severe disease is associated with more uncertainty. No interim analysis of response or toxicity data is planned. Side effects and possible side effects will be monitored continuously.

Cyclophosphamide is a well-known drug with a well-documented safety profile for several patient populations. Experiences with cyclophosphamide in the ME/CFS population is limited. In the event of SAEs, the study management in collaboration with the Safety Board will consider the need for unblinding in order to establish or rule out association with rituximab and, if relevant, characterise the SAE as a SUSAR. Should several SUSARs occur, the study management and Safety Board will consider discontinuing the inclusion of patients in the trial.

All safety data compiled in the study will be submitted as part of the secondary parameters for analysis, which will be reported in the final study results and end of study report.

Work-up before inclusion should exclude patients with suspected immunodeficiency disorders or ongoing active and relevant viral infections. However, as the cause of ME/CFS is as yet unknown, we cannot exclude the possibility that some patients may suffer an ongoing active viral infection which is undetectable at the work-up, and where the immunosuppressive effect of cyclophosphamide treatment theoretically could cause a clinical deterioration.

Side effects

A moderate dose of cyclophosphamide is usually administered as an uncomplicated 15 minute infusion preceded and followed by infusions of 500 ml saline, with a total treatment duration of approximately one hour.
Acute allergic reactions are very rare after cyclophosphamide infusions. Cyclophosphamide is an inactive substance which is metabolised in the liver, and does not cause necrosis in the case of extravasation. Infusions must be administered by a qualified nurse with a doctor present on site, in a facility equipped for medical emergencies.

Moderate doses are not expected to result in neutropenia, thrombocytopenia or significant hair loss, but patients may use a «cool cap» during infusion to prevent hair thinning. The most common side effect is nausea during the first 1 or 2 days after infusion.

Cyclophosphamide is a nitrogen mustard and an alkylating agent, and was introduced into the treatment of cancer patients more than 60 years ago. Cyclophosphamide is widely used in different cancers, both in combination with other chemotherapy agents and as monotherapy. Cyclophosphamide has a immunosuppressive effect and affects both humoral and cell-mediated immune response. Low to moderate doses of cyclophosphamide are also used in benign conditions such as rheumatoid arthritis, SLE, dermatomyositis, pemphigus, Wegener's granulomatosis and other systemic vasculitis, as well as in multiple sclerosis [28-30,38,39].

Cyclophosphamide is a well-known drug, and the safety profile for its use in malign diseases and autoimmune conditions is well-described. Alkylating agents have numerous significant side effects, such as bone marrow suppression, increased disposition to infections, toxic effect on the gonads and increased risk for developing secondary malignancies. Previously conventional treatment regimens with continuous oral cyclophosphamide over longer periods of time with high cumulative doses carried higher risks for long term toxicity on gonads and hematopoietic stem cells. The treatment regimen outlined in this protocol, with intravenous pulse therapy every four weeks with moderate doses and low cumulative doses, carry little risk of hematological side effects or infections.

However, Cyclophosphamide remains an antineoplastic agent with immunosuppresive effects, and it is important that the patient and treating physician discuss all possible side effects carefully, including the possibility for long term toxicity, so the patient can make an informed decision about participation in the trial. The possible serious side effects must be considered in the context of disease severity, duration and impact on quality of life for the individual patient.

The patients' hematological values will be monitored with blood samples before each treatemtn and additional blood tests at day 10-14 after the first two infusions, to check for neutropenia or thrombocytopenia during the nadir phase. If the patient develops an elevated temperature or sudden-onset major fatigue or new symptoms indicating an infection, the treating physician must be contacted promptly for blood tests to check for possible neutropenia. In case of neutropenia, the patient will be hospitalised according to procedures for treatment of neutropenic infections. The patient will receive written instructions on which symptoms to look out for and how to respond in case of an infection.

Alkylating agents may increase the risk of ovarian failure and premature menopause. The ris is higher in women over the age of 35. In a trial where the average age was 31 years, ovarian failure was registered in 13% [40]. Infertility can be seen in both men and women after use of alkylating chemotherapy, and in a trial where patients with lupus nephritis received six intravenous cyclophosphamide infusions, 13% reported persisting amenorrhea, while the risk for persisting amenorrhea in patients who received at least 15 doses was 39% [41]. Cyclophosphamide is teratogenic, and a serum pregnancy (HCG) test is performed before start of intervention. Patients are carefully informed of the importance of effective contraception during the study and for the following 12 months, both in the written patient

information letter and during the initial consultation. Male patients in part A of the study will be offered the option of cryopreservation of sperm before starting cyclophosphamide infusions.
Hemorrhagic cystitis can be caused by cyclophosphamide, and mainly occurs due to the urotoxic effects of the metabolite acrolein. The planned cumulative dose of between six and nine grams for 6 doses is safely below the estimated limit for the risk of developing both hemorrhagic cystitis and secondary bladder cancer [42]. Regardless, patients will be informed of the importance of staying well hydrated for 72 hours after each treatment, and a total of 1 litre of NaCl 0.9% is administered in conjunction with the cyclophosphamide infusion. A urine dipstick test is performed before each treatment to uncover any sign of microscopic hematuria.
Due to the low risk of hemorrhagic cystitis at the planned doses of cyclophosphamide, we will not administer Mesna (uromitexan) routinely. The effect of Mesna on bladder toxicity during cyclophosphamide treatment in rheumatic disease is uncertain, and results from this patient population are deemed relevant for the present study [42]. If study patients should develop hemorrhagic cystitis, they will be withdrawn from the study and not receive further treatments. Microscopic hematuria will be treated with Mesna (uromitexan) tablets.
Alopecia in low dose treatment is estimated at 20%. At the Dept. of Oncology, HUS, hair loss at the present doses is rarely seen, but patients may use a cool cap during treatment to further reduce the risk of hair loss.

A review article detailed the effects and side effects of intravenous pulse treatment with cyclophosphamide in 200 patients with multiple sclerosis, with relatively high cumulative doses of around 30 g. The article reported temporary amenorrhea in 60% and persisting amennorhea in 18% of women, temporary azoospermia in 60% of men, and reversible alopecia in 13% of patients. The risk of secondary malign disease is related to cumulative dose, and was reported at 1% within 10 years in this study [30].

In a trial where 112 patients with multiple sclerosis received intravenous pulse treatment with 700 mg/m^2 cyclophosphamide every four weeks for 12 months, followed by infusions every two months for another 12 months (18 infusions in total, cumulative dose approx. 25 g), malign disease is reported in four patients (3.6%). However, three out of the four had received other immunosuppressive treatment (azathioprine) before commencing cyclophosphamide treatment, and the trial concluded that the safety profile for intravenous pulse therapy with cyclophosphamide for treatment of non-malign diseases such as multiple sclerosis seems acceptable [43].

A study specifically investigating the risk of secondary malignancies in a French cohort of 354 patients with progressive multiple sclerosis who had been treated with cyclophosphamide, found that the estimated standardized incidence ratio for malign disease was equivalent to that of the general population for both men and women. The study concluded that there was no increased risk of secondary malignancies after cyclophosphamide treatment in multiple sclerosis [44].

Older cyclophosphamide regimens with continuous oral treatment with long-term exposure to drug and high cumulative doses (50-100 g) and a significantly increased risk of hemorrhagic cystitis and secondary malignancies including bladder cancer, are not considered relevant

when estimating the long-term toxicity from intravenous cyclophosphamide pulse therapy every four weeks.

The outlined treatment protocol proposes 6 infusions of intravenous cyclophosphamide and cumulative doses of approx. 6-9 g. The estimated risk of serious long-term toxicity other than the possible induction of menopause is low, but not negligible.

The justification for the trial lies in the recognition that the patients suffer from a condition which significantly affects their quality of life, and which has major implications for the patients, their dependents and for the society as a whole. A study shows that the quality of life of patients with ME/CFS is significantly reduced compared to that of patients with other chronic conditions such as rheumatoid arthritis, cardiac disease, pulmonary disease, diabetes or depression [45].
The patients will be given detailed information about safety aspects of the trial during the assessment consultation and in writing.

ETHICAL CONSIDERATIONS

ME/CFS is a serious affliction involving considerable suffering for the patient and great distress for relatives as well. No standardized, established medical treatment exists. Many young people are affected by the condition, with an estimated prevalence of approx. 0.1-0.2% of the population, i.e. between 5,000 and 10,000 patients in Norway and approx. 10,000,000 patients worldwide.

Considering that cyclophosphamide is an antineoplastic agent with immunosuppressive effects and potentially serious side effects as discussed above, its use in non-malign diseases must be well justified. Intravenous pulse therapy with cyclophosphamide is an established treatment in several autoimmune diseases, with an acceptable safety profile (see above). Several aspects suggest that ME/CFS could be a variant of an autoimmune disease with similar disease mechanisms as those found in some rheumatic systemic diseases, as outlined in the project description.
Even though ME/CFS is not characterised by organ damage, the symptoms experienced by patients are often debilitating. Risk of suicide is not negligible in patients with severe ME/CFS.
Our data from trials of rituximab in ME/CFS suggest that a patient subgroup have a disease which may be reversed by immunomodulatory treatment. However, a third of the ME/CFS patients in the completed rituximab trials did not report any clinically significant response after B-cell depletion. We plan to include patients who have not experienced response after rituximab infusions in part A of this study, in order to investigate if these patients may experience clinically relevant response after treatment with cyclophosphamide, which involves different mechanisms of immunosuppression. Patients who have relapsed after previous rituximab response may also need alternative treatment options due to such complications as the development of allergy to rituximab, anti-rituximab antibodies or hypogammaglobulinemia.

The outlined experiences from ME/CFS patients who received adjuvant chemotherapy due to breast cancer, as well as our preliminary pilot experience with cyclophosphamide as intravenous pulse therapy in ME/CFS, with 6 infusions and low cumulative dose, show a certain promise.

ME/CFS is associated with greatly reduced quality of life and frequently a need for care and assistance in daily activities, which is often handled by the patients' relatives. There is no established standard treatment, and we believe that is ethically justifiable to conduct a controlled trial in the form of an open phase II trial of cyclophosphamide as intravenous pulse therapy with 6 infusions in total, in order to evaluate effect, toxicity and feasibility.

Patients with a mild degree of ME/CFS will not be included in the study so that participation in part A is restricted to patients with severe, moderate or mild/moderate ME/CFS.
Male participants in part A may be referred to cryopreservation of sperm before start of treatment.
After the completion of the formal follow-up period (18 months) we will attempt to maintain contact with patients for up to five years in order to detect any long-term effects or toxicity.

ME/CFS affects a large patient population, many of whom are young people who are incapacitated from taking part in employment or education, and who suffer considerable distress. If the study should confirm that cyclophosphamide intervention is associated with clinically significant responses in ME/CFS, this could have great significance to a large number of patients. The cost of treatment is low; the acquisition cost for one dose of the drug is less than < 200 NOK.

The use of cyclophosphamide in ME/CFS carries a small, but not negligible risk (see detalis on side effects above), and we believe the risk is acceptable considering the possible benefit for the patients. The individual participant could potentially experience a clinical effect on their considerable symptom burden, which is often incapacitating with regards to social and family life, education or employment and general quality of life. The study will produce knowledge on the ME/CFS disease. We will systematically expand our existing biobank with blood samples from patients at baseline and at 6, 12 and 18 months' follow-up, and further biological spin-off studies on the biobank material will attempt to shed light on the pathogenic mechanisms behind ME/CFS.
The patients will be invited to a consultation where they will receive in-depth information and a written letter of information/declaration of informed consent, and will be given due time to consider before deciding on whether or not to participate in the study.

FUNDING

The study is investigator initiated. There is no external sponsor. The research team for ME/CFS at the Oncology Department at Haukeland University Hospital receives support from the Kavli foundation, mainly for research into the pathogenic mechanisms behind ME/CFS.
The Kavli foundation has funded a part time (50%) nursing position for twelve months in order to assist with the implementation of the trial. These funds will be transferred to the Clinical Research Unit at HUS who will be responsible for implementation and administration of treatment (part A) in collaboration with the study management at the Dept. of Oncology, HUS.
The drug cost is very low (< 200 NOK per dose), i.e. 1,200 NOK per patient for the whole course of treatment, and drug expenses will be covered by the ME/CFS research group at the Dept. of Oncology.

A trial-specific medical insurance will be taken out.
No financial compensation will be offered to the participants.

PUBLICATION

Co-authorship for the clinical study will be subject to participation in assessment and follow-up of patients. For supplementary biological analyses, physiological, neuropsychological or cognitive examinations, co-authorship in the main clinical study will depend on whether the data are used in the relevant publication. The order of authors in the publication will be decided by coordinating investigator Olav Mella.
The results – positive or negative – from the clinical study will be published in a reputable medical journal. Co-authorship and order of authors will comply with the Vancouver guidelines.
The study will be registered in ClinicalTrials.gov before study start date.
A final report will be submitted to the REC and NOMA.

APPLICATIONS FOR APPROVAL

Applications for approval will be sent to:
- The Regional Ethical Committee.
- The Biobank Register (extension of existing biobank).
- EudraCT.
- The Norwegian Medicines Agency.

REFERENCES

1. Nacul LC, Lacerda EM, Pheby D, Campion P, Molokhia M, et al. (2011) Prevalence of myalgic encephalomyelitis/chronic fatigue syndrome (ME/CFS) in three regions of England: a repeated cross-sectional study in primary care. BMC Med 9: 91.
2. Brenu EW, van Driel ML, Staines DR, Ashton KJ, Ramos SB, et al. (2011) Immunological abnormalities as potential biomarkers in Chronic Fatigue Syndrome/Myalgic Encephalomyelitis. J Transl Med 9: 81.
3. Broderick G, Fuite J, Kreitz A, Vernon SD, Klimas N, et al. (2010) A formal analysis of cytokine networks in chronic fatigue syndrome. Brain Behav Immun 24: 1209-1217.
4. Schutzer SE, Angel TE, Liu T, Schepmoes AA, Clauss TR, et al. (2011) Distinct cerebrospinal fluid proteomes differentiate post-treatment lyme disease from chronic fatigue syndrome. PLoS One 6: e17287.
5. Biswal B, Kunwar P, Natelson BH (2011) Cerebral blood flow is reduced in chronic fatigue syndrome as assessed by arterial spin labeling. J Neurol Sci 301: 9-11.
6. Duffy FH, McAnulty GB, McCreary MC, Cuchural GJ, Komaroff AL (2011) EEG spectral coherence data distinguish chronic fatigue syndrome patients from healthy controls and depressed patients - A case control study. BMC Neurol 11: 82.
7. Chang CM, Warren JL, Engels EA (2012) Chronic fatigue syndrome and subsequent risk of cancer among elderly US adults. Cancer 118: 5929-5936.
8. Carruthers BM, Jain AK, De Meirleir KL, Peterson DL, Klimas NG, et al. (2003) Myalgic encephalomyelitis/ chronic fatigue syndrome: clinical working case definition, diagnostic and treatment protocols. J Chronic Fatigue Syndr 11: 7-36.
9. Fluge O, Mella O (2009) Clinical impact of B-cell depletion with the anti-CD20 antibody rituximab in chronic fatigue syndrome: a preliminary case series. BMC Neurol 9: 28.
10. Fluge O, Bruland O, Risa K, Storstein A, Kristoffersen EK, et al. (2011) Benefit from B-Lymphocyte Depletion Using the Anti-CD20 Antibody Rituximab in Chronic Fatigue Syndrome. A Double-Blind and Placebo-Controlled Study. PLoS One 6: e26358.

11. Albright F, Light K, Light A, Bateman L, Cannon-Albright LA (2011) Evidence for a heritable predisposition to Chronic Fatigue Syndrome. BMC Neurol 11: 62.
12. Kessel A, Rosner I, Toubi E (2008) Rituximab: beyond simple B cell depletion. Clin Rev Allergy Immunol 34: 74-79.
13. Newton DJ, Kennedy G, Chan KK, Lang CC, Belch JJ, et al. (2012) Large and small artery endothelial dysfunction in chronic fatigue syndrome. Int J Cardiol 154: 335-336.
14. Sitia S, Tomasoni L, Atzeni F, Ambrosio G, Cordiano C, et al. (2010) From endothelial dysfunction to atherosclerosis. Autoimmun Rev 9: 830-834.
15. Murdaca G, Colombo BM, Cagnati P, Gulli R, Spano F, et al. (2012) Endothelial dysfunction in rheumatic autoimmune diseases. Atherosclerosis 224: 309-317.
16. Cooper DC, Tomfohr LM, Milic MS, Natarajan L, Bardwell WA, et al. (2011) Depressed mood and flow-mediated dilation: a systematic review and meta-analysis. Psychosom Med 73: 360-369.
17. Garcia X, Stein F (2006) Nitric oxide. Semin Pediatr Infect Dis 17: 55-57.
18. Coleman JW (2001) Nitric oxide in immunity and inflammation. Int Immunopharmacol 1: 1397-1406.
19. Steinert JR, Chernova T, Forsythe ID (2010) Nitric oxide signaling in brain function, dysfunction, and dementia. Neuroscientist 16: 435-452.
20. Steinert JR, Robinson SW, Tong H, Haustein MD, Kopp-Scheinpflug C, et al. (2011) Nitric oxide is an activity-dependent regulator of target neuron intrinsic excitability. Neuron 71: 291-305.
21. Murrough JW, Mao X, Collins KA, Kelly C, Andrade G, et al. (2010) Increased ventricular lactate in chronic fatigue syndrome measured by 1H MRS imaging at 3.0 T. II: comparison with major depressive disorder. NMR Biomed 23: 643-650.
22. Shungu DC, Weiduschat N, Murrough JW, Mao X, Pillemer S, et al. (2012) Increased ventricular lactate in chronic fatigue syndrome. III. Relationships to cortical glutathione and clinical symptoms implicate oxidative stress in disorder pathophysiology. NMR Biomed 25: 1073-1087.
23. Vermeulen RC, Kurk RM, Visser FC, Sluiter W, Scholte HR (2010) Patients with chronic fatigue syndrome performed worse than controls in a controlled repeated exercise study despite a normal oxidative phosphorylation capacity. J Transl Med 8: 93.
24. Vermeulen RC, Vermeulen van Eck IW (2014) Decreased oxygen extraction during cardiopulmonary exercise test in patients with chronic fatigue syndrome. J Transl Med 12: 20.
25. Brenu EW, van Driel ML, Staines DR, Ashton KJ, Hardcastle SL, et al. (2012) Longitudinal investigation of natural killer cells and cytokines in chronic fatigue syndrome/myalgic encephalomyelitis. J Transl Med 10: 88.
26. Hellsten Y, Nyberg M, Jensen LG, Mortensen SP (2012) Vasodilator interactions in skeletal muscle blood flow regulation. J Physiol 590: 6297-6305.
27. Green DJ, Jones H, Thijssen D, Cable NT, Atkinson G (2011) Flow-mediated dilation and cardiovascular event prediction: does nitric oxide matter? Hypertension 57: 363-369.
28. Brummaier T, Pohanka E, Studnicka-Benke A, Pieringer H (2013) Using cyclophosphamide in inflammatory rheumatic diseases. Eur J Intern Med 24: 590-596.
29. Riley P, Maillard SM, Wedderburn LR, Woo P, Murray KJ, et al. (2004) Intravenous cyclophosphamide pulse therapy in juvenile dermatomyositis. A review of efficacy and safety. Rheumatology (Oxford) 43: 491-496.
30. Patti F, Lo Fermo S (2011) Lights and shadows of cyclophosphamide in the treatment of multiple sclerosis. Autoimmune Dis 2011: 961702.
31. Fox LP, Pandya AG (2000) Pulse intravenous cyclophosphamide therapy for dermatologic disorders. Dermatol Clin 18: 459-473.
32. Zigmond AS, Snaith RP (1983) The hospital anxiety and depression scale. Acta Psychiatr Scand 67: 361-370.
33. Ware JE, Jr., Sherbourne CD (1992) The MOS 36-item short-form health survey (SF-36). I. Conceptual framework and item selection. Med Care 30: 473-483.

34. Myers C, Wilks D (1999) Comparison of Euroqol EQ-5D and SF-36 in patients with chronic fatigue syndrome. Qual Life Res 8: 9-16.
35. Loge JH, Kaasa S, Hjermstad MJ, Kvien TK (1998) Translation and performance of the Norwegian SF-36 Health Survey in patients with rheumatoid arthritis. I. Data quality, scaling assumptions, reliability, and construct validity. J Clin Epidemiol 51: 1069-1076.
36. Almeida GJ, Wasko MC, Jeong K, Moore CG, Piva SR (2011) Physical activity measured by the SenseWear Armband in women with rheumatoid arthritis. Phys Ther 91: 1367-1376.
37. Scheers T, Philippaerts R, Lefevre J (2011) Variability in physical activity patterns as measured by the SenseWear Armband: how many days are needed? Eur J Appl Physiol.
38. Fleischli ME, Valek RH, Pandya AG (1999) Pulse intravenous cyclophosphamide therapy in pemphigus. Arch Dermatol 135: 57-61.
39. Ntali S, Bertsias G, Boumpas DT (2011) Cyclophosphamide and lupus nephritis: when, how, for how long? Clin Rev Allergy Immunol 40: 181-191.
40. Mok CC, Ying KY, Ng WL, Lee KW, To CH, et al. (2006) Long-term outcome of diffuse proliferative lupus glomerulonephritis treated with cyclophosphamide. Am J Med 119: 355.e325-333.
41. Boumpas DT, Austin HA, 3rd, Vaughan EM, Yarboro CH, Klippel JH, et al. (1993) Risk for sustained amenorrhea in patients with systemic lupus erythematosus receiving intermittent pulse cyclophosphamide therapy. Ann Intern Med 119: 366-369.
42. Monach PA, Arnold LM, Merkel PA (2010) Incidence and prevention of bladder toxicity from cyclophosphamide in the treatment of rheumatic diseases: a data-driven review. Arthritis Rheum 62: 9-21.
43. Portaccio E, Zipoli V, Siracusa G, Piacentini S, Sorbi S, et al. (2003) Safety and tolerability of cyclophosphamide 'pulses' in multiple sclerosis: a prospective study in a clinical cohort. Mult Scler 9: 446-450.
44. Le Bouc R, Zephir H, Majed B, Verier A, Marcel M, et al. (2012) No increase in cancer incidence detected after cyclophosphamide in a French cohort of patients with progressive multiple sclerosis. Mult Scler 18: 55-63.
45. Nacul LC, Lacerda EM, Campion P, Pheby D, Drachler Mde L, et al. (2011) The functional status and well being of people with myalgic encephalomyelitis/chronic fatigue syndrome and their carers. BMC Public Health 11: 402.
