# Supplementary material for: Six-year follow-up of participants in two clinical trials of rituximab or cyclophosphamide in Myalgic Encephalomyelitis/Chronic Fatigue Syndrome
Source: PLoS One. 2024 Jul 23;19(7):e0307484. doi: 10.1371/journal.pone.0307484 (PMC11265720; doi:10.1371/journal.pone.0307484)
Supplement: S6 File — (PDF) [file pone.0307484.s007.pdf]

Region: REC North
Our date: 26.04.2021
Our reference: 25331
Your reference: REK nord

Øystein Fluge

25331 B-lymphocyte depletion using the anti-CD20 antibody rituximab in Chronic Fatigue Syndrome

Responsible institution: Helse Bergen HF - Haukeland University Hospital

Applicant: Øystein Fluge

REC evaluation
We refer to your application for project amendment regarding the above mentioned research project, date of receipt 15 April 21. The application has been processed by the REC North administration on delegated authority by the committee as governed by the Regulation of research ethics § 7, first paragraph, third sentence. The application has been assessed with legal authority in the Health Research Act, § 11.

The applicants seek to re-contact the participants in order to assess patients' symptoms and disease status six years after inclusion in the trial. A letter of information and consent will be sent to the participants.

According to the application for project management, the purpose is as follows:

To examine the course of disease over the last four years, including an assessment of which patients have experienced improvement, a stable condition or worsening of their health status.

These data will be analysed against variables such as:

disease severity
disease duration
sex
age
whether the disease was triggered by an infection

Data will also be analysed with relation to group randomization to intervention or control group.

Data will be collected through questionnaires that will be mailed to participants along with the letter of information and consent.

Questionnaires include one form that has been designed by the project specifically for this project amendment, where participants are asked to report their function level and employment status as well as any new medical conditions and other treatments.

Additional questionnaires are DSQ, SF36 and a study-specific, patient-reported questionnaire, which was previously completed by participants during the completed trial.

On delegated authority, the administration has made the following

Decision

Pursuant to the Health Research Act, § 11, the project amendment is approved.

The project is approved until end date 31 December 2025 as requested.

For documentation purposes, the data must be stored for five years after project end date. Any access to project data during the storage period should be for control purposes only, and the project data will not be available for the project. The project manager and responsible institution are responsible for the storage of data during this period in an indirectly identifiable manner, i.e. separated in a data file and an ID code file.

After the five year period, the information must be deleted or anonymized. The committee points out that anonymization requires more than simply deleting the ID code; refer to the Data Protection Authority guidelines on anonymization techniques.

Please also note that with reference to the Personal Information Act, there must be a legal basis for data processing in accordance with the Personal Data Protection Ordinance. This aspect must be handled by the responsible institution.

Yours sincerely,

May Britt Rossvoll
head of administraton

Lill Martinsen
senior adviser

Right of appeal

You may appeal the committee's decision, cf. the Public Administration Act § 28 et seq. The appeal may be directed to REC North. The appeal deadline is three weeks from the receipt of this letter. If the decision is upheld by REC North, the appeal will be forwarded to the national research ethics committee for medicine and health sciences (NEM) for a final decision.

Region: REC North
Our date: 25.10.2022
Our reference: 9769

Øystein Fluge

Project application: Cyclophosphamide in Myalgic encephalopathy/ Chronic Fatigue Syndrome (ME/CFS)
Application number: 2014/1672
EudraCT number: 2014-004029-41
Responsible institution: Helse Bergen HF - Haukeland University Hospital.
Collaborative responsible institutions: Helse Bergen HF - Haukeland University Hospital

Project application: Amendment approved

Applicant's description

The objective of the study is to assess whether cyclophosphamide infusions every four weeks are associated with clinically significant responses and acceptable toxicity in ME/CFS patients, and whether such treatment is feasible in patients with severe and very severe ME/CFS. The primary endpoint will be based on patients' self-report of symptom development.

The application concerns part A: an open phase II trial of up to 40 patients with ME/CFS (excluding mild severity) with six infusions of cyclophosphamide every four weeks, 12 months follow-up.

If part A has a response rate of at least 40%, part B will follow: A descriptive study of up to 20 patients with severe and very severe ME/CFS, following said treatment schedule, but where follow-up and intervention are administered in collaboration with patients' local health services.

We refer to your application on project amendment for the above-mentioned research projected, date of receipt 20 October 2022. The application has been processed by the REC North administration on delegated authority by the committee as governed by the Regulation of research ethics § 7, first paragraph, third sentence. The application has been assessed with legal authority in the Health Research Act, § 11.

The applicants seek to administer a follow-up investigation in the project, where they will contact the 40 project participants in order to assess symptoms and ME/CFS disease status approximately seven years after inclusion in the trial.

A follow-up investigation after five years, which involved telephone interviews and activity registration using an acitivity monitor was approved by REC and conducted in 2019.

This follow-up investigation will be based solely on patient-reported questionnaire data.

The questionnaires will be sent to the participants by mail along with a letter of information. Prior to the letter, an SMS will be sent to advise the participants to expect the follow-up questionnaires. Further, applicants plan to send two SMS reminders to participants who have failed to respond, after 4 and 8 weeks respectively.

Applicants state that data will be treated in a de-identified manner and according to the trial-specific data management plan.

REC has no objections to the amendment.

On delegated authority, the administration has made the following

Decision

Pursuant to the Health Research Act, § 11, the project amendment is approved.

The amendment is approved provided that the project is conducted as described in the application, the application for amendment, the updated protocol and the provisions that follow from the Health Research Act including regulations. Conditions that have been set in connection with previous approvals of the project, also still apply.

End of study notification

Project manager must send an end of study notification to REC on the relevant form via the REC portal no longer than six monts after end of study date, 31.12.2025, as stated in the Health Research Act § 12. If the project is not initiated or completed, this must also be reported on the end of study notification form.

Amendment application

If you wish to make substantial amendments to the objectives, method, time frame or organisation, the project manager must send an application for amendment via portal on the relevant form to REC, cf. The Health Research act § 11.

Klageadgang

You may appeal the REC's decision, cf. the Public Administration Act § 28 et seq. The appeal must be sent using the relevant form in the REC portal. The appeal deadline is three weeks from the receipt of this letter. If the decision is upheld by REC, the appeal will be forwarded to the national research ethics committee for medicine and health sciences (NEM) for a final decision, cf. The Research Ethics Act § 10 and the Health Research Act § 10.

Yours sincerely,

May Britt Rossvoll
Head of administration

Ragnhild Hageberg
Senior adviser

Copy to:
Helse Bergen HF - Haukeland University Hospital
The Norwegian Medicines Authority
