# Supplementary material for: Six-year follow-up of participants in two clinical trials of rituximab or cyclophosphamide in Myalgic Encephalomyelitis/Chronic Fatigue Syndrome
Source: PLoS One. 2024 Jul 23;19(7):e0307484. doi: 10.1371/journal.pone.0307484 (PMC11265720; doi:10.1371/journal.pone.0307484)
Supplement: S7 File — (PDF) [file pone.0307484.s008.pdf]

Supplemental Methods, Statistical analyses

Syntax: General Linear Model repeated measures

```
GLM SF36PF_0 SF36PF_18 SF36_PF_6y BY Trial WITH Age_inclusion Sex Center ME_severity2
  /WSFACTOR=time 3 Simple(1)
  /MEASURE=SF36PF
  /CONTRAST(Trial)=Simple(1)
  /METHOD=SSTYPE(3)
  /PLOT=PROFILE(time*Trial) TYPE=LINE ERRORBAR=CI MEANREFERENCE=NO YAXIS=AUTO
  /EMMEANS=TABLES(OVERALL) WITH(Age_inclusion=MEAN Sex=MEAN Center=MEAN
ME_severity=MEAN)
  /EMMEANS=TABLES(Trial) WITH(Age_inclusion=MEAN Sex=MEAN Center=MEAN
ME_severity2=MEAN)COMPARE
    ADJ(LSD)
  /EMMEANS=TABLES(time) WITH(Age_inclusion=MEAN Sex=MEAN Center=MEAN
ME_severity=MEAN)COMPARE
    ADJ(LSD)
  /EMMEANS=TABLES(Trial*time) WITH(Age_inclusion=MEAN Sex=MEAN Center=MEAN
ME_severity=MEAN)
  /PRINT=DESCRIPTIVE ETASQ PARAMETER
  /CRITERIA=ALPHA(.05)
  /WSDESIGN=time
  /DESIGN=Age_inclusion Sex Center ME_severity Trial
```

SPSS Analysis code for General Linear Model repeated measures, as example for SF-36 Physical Function (SF36PF) assessing the difference between trial groups in repeated measures through follow-up (from baseline to 18 months to 6 years).
Time was included as within-subject factor (3 levels for the outcome measure). Between-subject was trial group (RituxME versus CycloME). The model utilized Type III sum of squares.

The interaction time*trial group was included as predictor.
For all General Linear Model analyses with 3 repeated measures, Mauchly's test was significant ($p<0.001$) indicating violations of the sphericity assumption, and Greenhouse-Geisser corrections were used.
For calculation of P-value for the interaction term time*trial group, we included as covariates in the model: age at inclusion, sex, baseline ME/CFS severity (3 levels: Mild/mild-moderate; Moderate; Moderate/severe-severe), and study center (five centers in the RituxME trial, one center in the CycloME trial).

Estimated marginal means were calculated for the variables trial group, time, and interaction time*trial group. The plots for trial group versus time (Figure 2) included 95%

confidence intervals. Output included descriptive statistics, parameter estimates, and estimates of effect sizes including the contrasts between trial groups, i.e. differences between trial groups for the averaged outcome measures with 95% confidence intervals.

Simple contrasts were used in time domain for the interaction term time*trial group, to assess changes in difference between treatment groups at each time level compared to baseline. All analyses were performed using IBM SPSS Statistics ver. 28 (IBM Corp., Armonk, NY). Figures were made in Graphpad Prism ver.10 (GraphPad Software, La Jolla, CA).

Syntax, Logistic regression analysis

```
LOGISTIC REGRESSION VARIABLES SF36PF_6y_vs70
  /METHOD=BSTEP(COND) Age_inclusion Sex Center Trial SF36PF_0
  /CONTRAST (Sex)=Simple(1)
  /CONTRAST (Center)=Simple(1)
  /CONTRAST (Trial)=Simple(1)
  /PRINT=SUMMARY CI(95)
  /CRITERIA=PIN(0.05) POUT(0.10) ITERATE(20) CUT(0.5).
```

Logistic regression analysis was performed with SF-36 Physical Function (SF-36 PF) with cut-off $<$ 70 versus $>=$ 70 as the dependent variable. Method was backwards stepwise (conditional). Age at baseline, sex, study center (5 centers in RituxME and one center in CycloME), baseline SF-36 PF (scale 0-100) and clinical trial (RituxME versus CycloME) as the predictor variables.
